# Usability and Perception of a Wearable-Integrated Digital Maternity Record App in Germany: User Study

**DOI:** 10.2196/50765

**Published:** 2023-12-15

**Authors:** Michael Nissen, Carlos A Perez, Katharina M Jaeger, Hannah Bleher, Madeleine Flaucher, Hanna Huebner, Nina Danzberger, Adriana Titzmann, Constanza A Pontones, Peter A Fasching, Matthias W Beckmann, Bjoern M Eskofier, Heike Leutheuser

**Affiliations:** 1 Machine Learning and Data Analytics Lab, Friedrich-Alexander Universität Erlangen-Nürnberg, Erlangen, Germany; 2 Department of Social Ethics, University of Bonn, Bonn, Germany; 3 Department of Gynecology and Obstetrics, Erlangen University Hospital, Friedrich-Alexander Universität Erlangen-Nürnberg, Erlangen, Germany

**Keywords:** maternity log, maternity logbook, log, logbook, experience, experiences, attitude, attitudes, opinion, opinions, perception, perceptions, perspective, perspectives, pregnancy record, personal health record, PHR, health records, health record, feature, features, develop, development, maternity record, electronic, digital, paper hand-held record, mHealth, mobile health, app, apps, application, applications, smartphone, smartphones, wearable, wearables, usability, pregnant, pregnancy, maternal, maternity, electronic maternity record, pregnancy app, data sharing, privacy, online search, searching, information behavior, information behaviour, information seeking

## Abstract

**Background:**

Although digital maternity records (DMRs) have been evaluated in the past, no previous work investigated usability or acceptance through an observational usability study.

**Objective:**

The primary objective was to assess the usability and perception of a DMR smartphone app for pregnant women. The secondary objective was to assess personal preferences and habits related to online information searching, wearable data presentation and interpretation, at-home examination, and sharing data for research purposes during pregnancy.

**Methods:**

A DMR smartphone app was developed. Key features such as wearable device integration, study functionalities (eg, questionnaires), and common pregnancy app functionalities (eg, mood tracker) were included. Women who had previously given birth were invited to participate. Participants completed 10 tasks while asked to think aloud. Sessions were conducted via Zoom. Video, audio, and the shared screen were recorded for analysis. Task completion times, task success, errors, and self-reported (free text) feedback were evaluated. Usability was measured through the System Usability Scale (SUS) and User Experience Questionnaire (UEQ). Semistructured interviews were conducted to explore the secondary objective.

**Results:**

A total of 11 participants (mean age 34.6, SD 2.2 years) were included in the study. A mean SUS score of 79.09 (SD 18.38) was achieved. The app was rated “above average” in 4 of 6 UEQ categories. Sixteen unique features were requested. We found that 5 of 11 participants would only use wearables during pregnancy if requested to by their physician, while 10 of 11 stated they would share their data for research purposes.

**Conclusions:**

Pregnant women rely on their medical caregivers for advice, including on the use of mobile and ubiquitous health technology. Clear benefits must be communicated if issuing wearable devices to pregnant women. Participants that experienced pregnancy complications in the past were overall more open toward the use of wearable devices in pregnancy. Pregnant women have different opinions regarding access to, interpretation of, and reactions to alerts based on wearable data. Future work should investigate personalized concepts covering these aspects.

## Introduction

Home-based maternal records, also referred to as pregnancy logs, maternity records, or hand-held paper records, store women’s medical information during pregnancy. These records contain various information, for example, about vaccinations, blood pressure, or ultrasound examinations [1]. They are used in more than 163 countries and greatly vary in design and content [2]. Home-based maternal records have been shown to provide advantages. They establish a better connection between caregivers and pregnant women, an improved sense of women’s empowerment, increased knowledge, family involvement, and continuity of care [3,4].

Digitalization has the potential to alleviate existing pitfalls of home-based maternal records. In a previous study, 36% of women involved in one trial stated that they forgot their records during at least one visit to their caregiver [5]. This and other problems can be solved by digital maternity records (DMRs). DMRs can improve information transfer between general practitioners, gynecologists, midwives, and hospitals [6]. Furthermore, clarity and documentation efforts can be improved while avoiding media discontinuities, that is, changes between communication media, such as (manual) data transfer from fax to paper or digital [7].

Several studies have implemented and investigated the effect of DMRs: Providing maternity data to pregnant women on flash drives resulted in advances in patient empowerment, satisfaction, and safety [8]. A digital pregnancy information and journaling tool could increase patient activation [9], although this may decrease over time compared to paper-based tools [10]. DMRs are mostly perceived as positive [11,12], although confidentiality, privacy, and data control are of concern [13]. A study in the Netherlands [14] found no effect on quality of care or outcomes.

The openness toward the use of wearable devices in maternal care is mixed [15,16]. Motivation to use wearable devices increases if the use is associated with positive outcomes [16]. At the same time, pregnant women state that incorrect or out-of-normal measurements from wearables can be a source of anxiety [16]. A trial that integrated wearables into routine maternal care reported that most participants continued to use the devices through pregnancy and after birth [17].

Patient-centered design transfers the principles of user-centered design (UCD) to the health care domain. UCD aims to increase the chance of user acceptance through regular and iterative inclusion of users in the development process [18,19]. Evidence on usability is crucial for the implementation of new apps in maternal health care [20].

Some studies on DMRs have investigated usability: Shaw et al [21] provided pregnant women with access to websites containing antenatal health information. One group received access to their individual antenatal health record, the other to general pregnancy health information. Users provided with personalized information logged in 6 times as often as users receiving general antenatal health information. Participants in both groups were highly satisfied with the website itself. Chang et al [22] developed a system integrating web-based maternity records, including explanations about individual tests; self-care journals for weight, blood pressure, movement and contraction tracking; and educational features. A survey administered to 68 pregnant participants revealed that the pregnancy calculator, estimated date of birth, and body calculator were particularly relevant for pregnant women, and 80.9% of participants stated the system was useful for their pregnancy. In a previous study, we investigated the use of a DMR interface by physicians and midwives [23]. The completion time for DMR data entries was about 30% higher, while the average number of errors was lower, compared to the analogue version.

However, most studies investigating usability aspects in DMRs have used postuse questionnaires. While this provides general usability metrics, it provides no insights on users’ thought processes and behavior. To date, no study has examined the use of a DMR app for pregnant women with live user testing. The effect of displaying wearable data in a pregnancy app has also not been investigated to date. Thus, this work aimed to investigate the usability and perception of a medically guided DMR integrating wearable data by conducting a usability study using a DMR prototype app, think-alouds, and semistructured questionnaires.

## Methods

This work used a mixed methods approach consisting of think-alouds during several usability tasks followed by a semistructured interview.

### Ethical Considerations

The study was approved by the ethics committee of Friedrich-Alexander Universität Erlangen-Nürnberg (106_13 B). The participants provided informed consent to participate.

### Concept and Features

A novel DMR app was developed jointly by obstetricians, ethicists, and computer scientists. This app combined four aspects: (1) DMR functionality, that is, providing users with their personal antenatal care data, (2) the integration of wearable devices, (3) medical trial functionality to deliver questionnaires for this study and other prospective mobile and ubiquitous health studies, and (4) additional features known from commercial pregnancy apps (general information section, week-by-week information, mood tracker).

First, the DMR functionality was developed in close alignment with the official and standardized paper-based German home-based maternal record (Mutterpass). For each section of the Mutterpass, a respective digital page was created (“Lab results,” “Previous pregnancies,” “Consultation,” “Anamnesis,” “Special findings,” “Date estimation,” Gravidogram,” “Hospitalizations,” “Cardiotocography,” “Ultrasound,” “Epicrisis”). We refrained from implementing potential improvements related to digitalization or making other major changes, as the official German DMR (E-Mutterpass) currently rolled out is in line with our approach [24]. Second, wearable devices are integrated by displaying heart rate, sleep, and blood pressure data. Third, study functionality is organized around a “My tasks” page. Each category offers visualization options for all data, as well as monthly, weekly, and textual representations. Fourth, the implementation of selected functionalities typically found in pregnancy apps, including a mood tracker, information section, and week-by-week information, aims to improve the overall attractiveness of the app.

### Development

**Figure 1.**
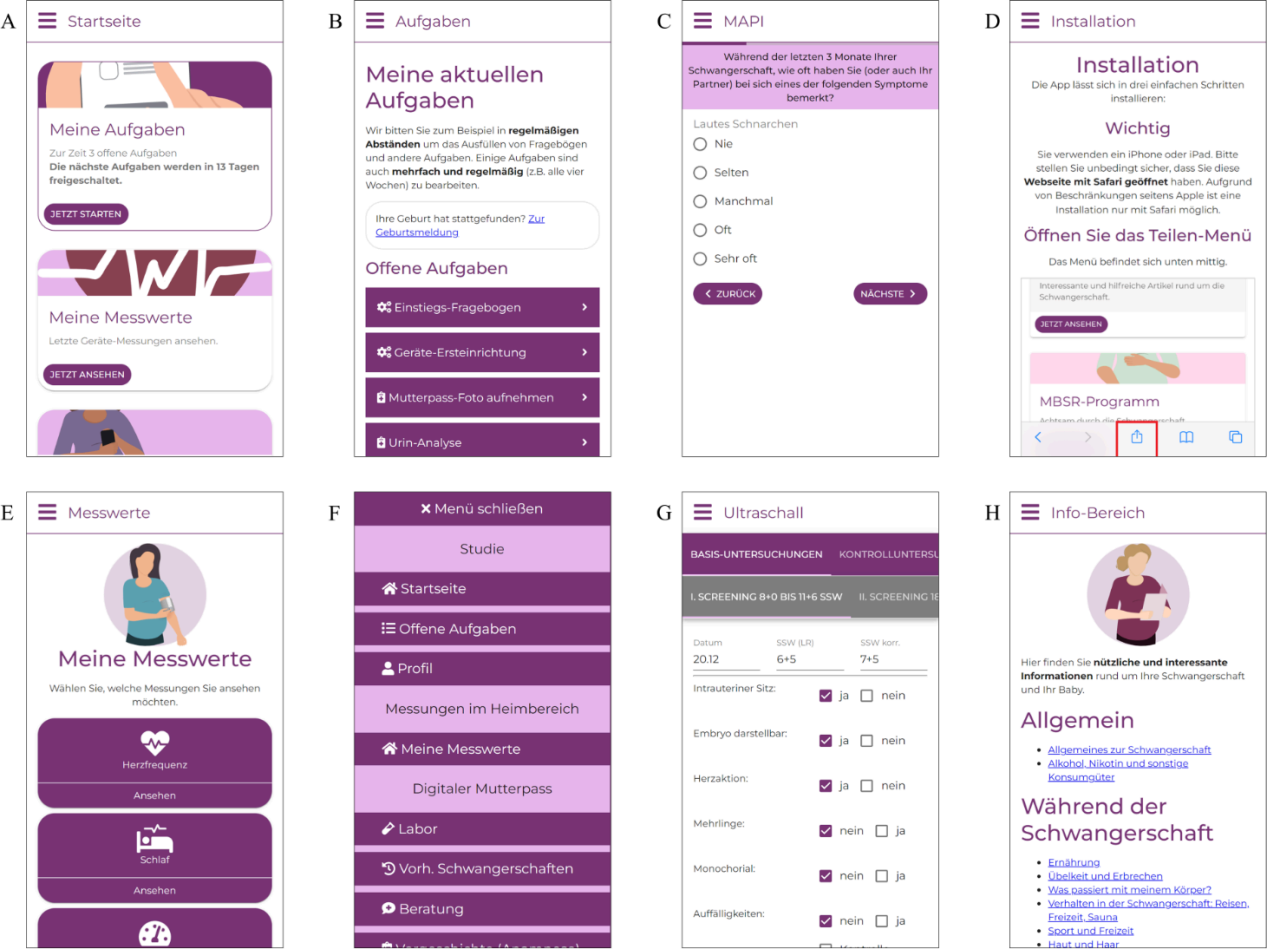
Example screenshots of the developed app. (A) “Start,” (B) “My tasks,” (C) “Mapi questionnaire,” (D) “Installation guide,” (E) “My measurements,” (F) “Main menu,” (G) “Ultrasound section,” and (H) “Information” sections. The app is intended for a German audience and was thus developed in German. A retrospectively translated English version of these screenshots is provided in Multimedia Appendix 1.

The app is a progressive web app (PWA) developed using ReactJS (Facebook Inc) as frontend framework. The user interface uses components from MUI (Material-UI SAS). The backend uses the *carecentive* framework [25]. *Carecentive* is implemented using Node.js (OpenJS Foundation), relying on Express.js (OpenJS Foundation) for web server functionality and MySQL (Oracle Corp) as the database. Screenshots of the developed app are shown in Figure 1. The app was developed in German.

### Recruitment

A flyer for participant recruitment was designed and was distributed using the local university department’s mailing list, student Facebook groups, local businesses displays, and personal contacts. The flyer included information about the prerequisites for participation. These included having reached the legal age of maturity (18 years), having had one or more pregnancies in the recent past, and having German and English language skills. We did not use a fixed time threshold for the time since the last pregnancy, as participant recruitment for similar studies with such fixed constraints proved to be difficult in the past. Interested individuals were asked to contact the study advisor by email or phone; this contact information was also included in the flyer.

### Study Setup

The study was conducted from December 7, 2021, to January 11, 2022. Germany experienced a significant number of COVID-19 infections during this timeframe. After careful evaluation, we determined that conducting a lab study with in-person attendance was unreasonable, as our study aims could still be achieved through virtual meetings. Zoom (version 5.8.1; Zoom Video Communications, Inc) was used for this purpose.

### Procedure

After expressing their interest in the study, participants were sent a Zoom link along with the respective consent forms. After participants provided informed consent, the study advisor started audio and video recording. Following this, participants received study instructions as a PDF document by email. This document led participants through the procedure alongside the study advisor. It comprised questionnaires and instructions for individual tasks.

Afterward, a PDF questionnaire for demographic data (age, number of previous pregnancies, months since last pregnancy, education level, household income, high-risk pregnancy, and familiarity with the German Mutterpass) and technical affinity [26] (enthusiasm, expertise, and positive and negative attitude) was filled out. A short video about pregnancy in gestational week 25 was shown to increase participants’ pregnancy immersion [27].

The study advisor shared the screen of a smartphone (Huawei Y7 2017; Android 7.0) via Zoom. A tool that mirrors a smartphone screen on the computer was used for this purpose (Scrcpy version 1.21; Genymobile). Participants were able to control the full mobile phone (not only a browser), including the ability to change phone settings or install apps. The screen was shared with the participant, and interactive use of a keyboard and mouse was enabled. Participants were now able to interact with the app in the same way as if it was used on their computers. This was necessary to record the interactions of the participant with the app for later evaluation. The installation of screen recording software and consecutive transfer of files to the study coordinators was previously deemed unfeasible due to the required technical expertise and significant effort of participants.

Participants were asked to complete 10 tasks and asked to freely express their feelings and thoughts during app use (eg, provide “think-alouds”). The tasks and their respective aims are shown in Table 1. Example screenshots for each task can be found in Multimedia Appendix 2. Following this, users completed the User Experience Questionnaire (UEQ) [28] and System Usability Scale (SUS) [29] in a digital form directly in the app. A semistructured interview about perceptions and personal preferences regarding information search in pregnancy, wearable device use, integration into maternal care, digitalization potential, and research data sharing concluded the study.

**Table 1.** List of study tasks.

Task ID	Description	Specific aim(s)	Evaluation of success, time, and errors?
1	Create a new user and login	Test login and registration procedure	Yes
2	Install the app on the mobile phone	Test progressive web app installation	Yes
3	Free exploration	Assess general perception and initial thoughts	No
4	Complete Multivariable Apnea Prediction Index [30]	Test questionnaire interface by using a specific questionnaire as an example	Yes
5	Explore ultrasound section	Test ultrasound section, particularly the Material-UI tabs	No
6	Find remarks on second ultrasound checkup	Test design of digital maternity record section	Yes
7	Explore visualization options	Test wearable data display and presentation	No
8	Find a heart rate value	Test wearable data display and presentation	Yes
9	Find depression information	Test design of information section	No
10	Enter and save information in mood journal	Test design of mood journal	Yes

### Evaluation

SUS, UEQ, and think-aloud remarks were used to evaluate usability. General perception was assessed through the think-aloud and semistructured interview at the end. Personal preferences and habits on online information searching, wearable data presentation and interpretation, at-home examination, and willingness to share data for research purposes were also assessed through the semistructured interviews at the end of the study.

Task completion times (TCTs), task success, errors, and self-reported (free text) feedback were recorded to assess effectiveness and efficiency. TCTs were extracted from video recordings. Task success was defined as all actions being clearly identified and completed, while failure was defined as at least one of the actions composing the task failing once. Errors were categorized as slips (unintended actions such as typos or accidental clicks) and mistakes (wrong actions thought to be correct, such as clicking on a nonclickable item) [31]. GOMS (Goal, operators, methods, and selection rules) modeling using Cogulator (Mitre Corp) was used to estimate reference times. GOMS modeling analyzes and aims to predict user interaction with computer systems. It helps designers, developers, and researchers to understand user behavior. User tasks are divided into goals. Each of the goals is achieved by solving subgoals in a divide-and-conquer approach [32]. In the context of this work, a reference time was estimated by dissecting each task into different subtasks. Each of these subtasks (eg, typing a text, pointing and clicking on an item, processing information) was associated with a certain time.

Oral remarks and comments were aggregated into 3 categories: feature requests, comments about user experience, and identified bugs.

## Results

### Participants

A total of 14 participants scheduled appointments for study participation. Two (P6, P7) did not fulfill the inclusion criteria. The screen-recording data of one participant (P3) could not be saved due to technical issues. This participant was thus excluded. Finally, 11 participants were included in the evaluation.

Participants were aged 34.6 (SD 2.2) years, had 1.8 (SD 0.98) previous pregnancies, and had their last delivery 33.2 (SD 24.5) months previously. Education level (n=1: job training; n=3: bachelor’s degree; n=1: master’s degree; n=6: PhD) and monthly household gross income varied (n=1: €3000-€6000; n=3: €6000-€9000; n=3: €9000-€12,000; n=4: >€12,000; a conversion rate of €1=US $1.13 applies). Most participants were native German speakers (n=9). The remaining 2 participants reported basic (n=1) and advanced (n=1) German language skills.

### Task Completion Time

**Figure 2.**
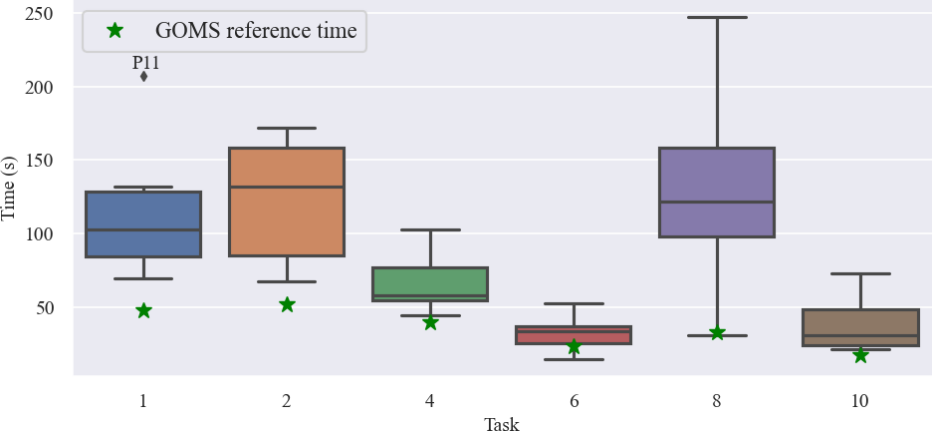
Task completion times for each task. See Table 1 for details on the individual tasks. GOMS (goal, operators, methods, and selection rules) modeling using Cogulator was performed to estimate reference times. No times were measured for explorative tasks (tasks 3, 5, 7, 9).

The task completion times and GOMS reference times of each task are shown in Figure 2. As outlined in Table 1, no times were measured for explorative tasks.

### Errors and Task Success

Recorded errors per task and overall task success rates are shown in Table 2.

**Table 2.** Total error count and task success per task. No times were measured for explorative tasks (tasks 3, 5, 7, and 9).

Task ID	Errors, n	Successfully completed tasks (n=11), n (%)
1	1	2 (18)
2	17	11 (100)
4	1	11 (100)
6	4	11 (100)
8	8	5 (46)
10	3	11 (100)

### Usability Questionnaires

**Figure 3.**
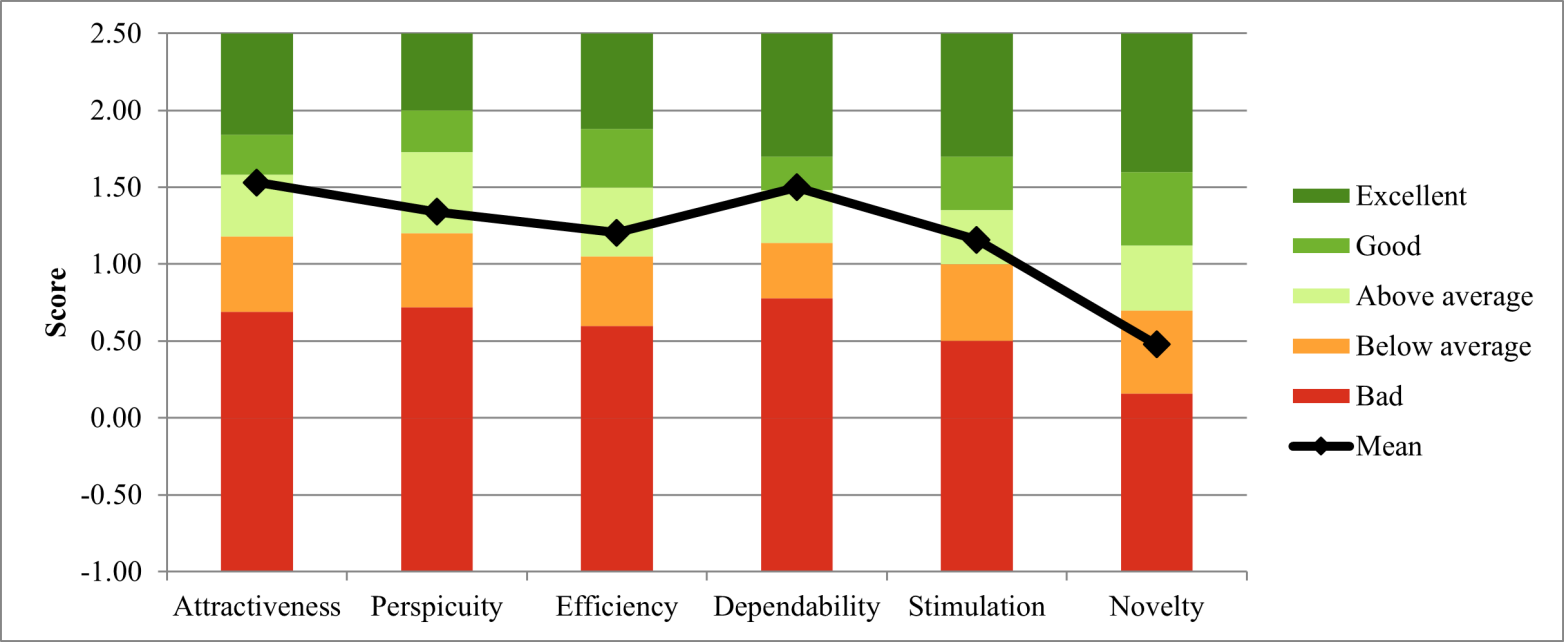
User Experience Questionnaire (UEQ) results. The app was rated as “above average” in most areas. The UEQ scales range from −3 to +3. The graphic was derived from the official UEQ evaluation benchmark tool, which crops ranges to improve readability.

The app received a mean SUS score of 79.09 (SD 18.38). According to the UEQ, participants rated the app’s attractiveness, perspicuity, efficiency, and stimulation as “above average.” It was rated as “good” in dependability and “below average” in novelty (Figure 3).

### Oral Think-Aloud Remarks and Comments

Participants requested a total of 23 features, of which 16 were unique (Table 3). The study identified 6 unique bugs (Table 4). Participants made 35 additional usability-related comments regarding 25 unique items (Table 5).

**Table 3.** Feature requests as stated by users during the study.

Description	Context	Users, n
Interactive graphs: zoom, pan, hover	Data visualization	4
Upload ultrasound picture	Ultrasound	3
Emergency contact phone numbers	General	2
Send recommendations based on mood trend	Journal	2
Gravida and para values updated automatically	Maternity logbook	1
Contextual explanation of concepts that appear in the app	General	1
Contextual explanation of pregnancy concepts in the app	General	1
Provide information about size and weight of the baby	General	1
Information about items needed when giving birth	Information	1
Information on health professionals involved during pregnancy	Information	1
Reuse information from previous pregnancies	Maternity logbook	1
Overview of scheduled appointments as in the paper version	General	1
Show a questionnaire to enter basic data at first app use	General	1
Show visualization of mood trend	Journal	1
Support multiple pregnancies	Maternity logbook	1
Ultrasound curves	Ultrasound	1

**Table 4.** Bugs encountered by users during the study.

Description	Users, n
Automatic logout after some time	7
Scrolling up to the top refreshes the app	6
Installation did not work	1
Opening tasks menu does not delete completed tasks	1
Page reload for unknown reason	1
Questionnaire was not sent	1

**Table 5.** Other usability-related comments made by users during the study.

Description	Context	Users, n
Show meaning of abbreviations	General	4
Too much scrolling in the app	General	3
Side menu: too much information	General	3
Unclear why the app needs to be installed	App installation	2
Confusing that the Mutterpass data is editable	Maternity logbook	2
Side menu: avoid scrolling up each time to access side menu	General	2
Heart frequency plots without labels	Data visualization	1
Show the gravidogram version in a user-friendly way	Maternity logbook	1
Visualization options: not easy to find	Data visualization	1
Side menu: use collapsible structure	General	1
Side menu: make it sticky to avoid scrolling up to open it	General	1
Side menu: colors make it unclear which parts are clickable	General	1
Show time estimated to finish a questionnaire	General	1
Show previous ultrasound examinations first by default	Ultrasound	1
Show reference values in plot	Data visualization	1
Heart frequency plots without legends	Data visualization	1
Reduce the amount of information on each screen	General	1
Reduce number of needed clicks in questionnaires	General	1
Horizontal scrolling tabs not discoverable	General	1
Hide technical information from the Mutterpass by default	Maternity logbook	1
Color code open tasks if done or in progress	Open tasks	1
Use smaller icons in main screen to reduce scrolling	General	1
Due date estimation: too much information	Maternity logbook	1
Journal option not discoverable	Journal	1
Visualization options: text would look better as a table	Data visualization	1

### Semistructured Interviews

The following paragraphs summarize the findings from the semistructured interviews. A more detailed tabular summary is provided in Multimedia Appendix 3.

#### Information Seeking and Online Research

Participants were initially asked about their preferred information depth for medical pregnancy information. For example, they were asked whether they preferred only basic information, as they may feel overwhelmed, or wanted to know “all pregnancy details.” Participants answered either that they had no problem during their pregnancy and thus could not answer the question (1/11), did not prefer deep information (1/11), relied on their physician to deliver adequate information (3/11), felt overwhelmed by information when things were not in order (1/11), or wanted to know all information (5/11). Almost half (5/11) of the participants searched online for information. Three explicitly mentioned that they do not perceive online forums or blogs as trustable. Apps were mentioned 3 times, although one participant said that she ultimately did not use them, as she did not want to pay for them. The reliability of sources, such as apps officially published by a government authority, was a reoccurring topic (4/11). Some participants stated they used books or scientific papers (3/11).

#### Wearables and Wearable Data Interpretation

Participants were invited to discuss the use of wearables during pregnancy, as well as the resulting data presentation (such as vital parameters) and interpretation. Two participants stated they would use wearables as part of regular prenatal care, with one having had a situation in the past where the technology would have been helpful. Almost half (5/11) mentioned that they would only use wearables if required to do so by their physician. One participant mentioned that the use of wearables might be helpful if risk factors were present (such as underweight or obesity). Two participants explicitly stated that they would not use wearable devices. Privacy and cyberchondria concerns were mentioned by 2 participants each.

Regarding the presentation and communication of data-derived information in the app, 1 participant stated that the full data must be accessible to the women. On the contrary, 2 mentioned that the data should only be interpreted by the physician. When asked about how users should be informed about detected anomalies in the recorded data, 6 women stated that they would like to receive a notification in these cases. Out of these 6, 1 said that a physician visit should be enforced after receiving such a notification, another requested immediate feedback to assist with the problem (eg, breathing exercises), 1 stated that data should be generally hidden as she did not understand it, and 2 stated that these notifications should only appear upon severe anomalies.

In terms of data visualization, 2 participants mentioned that this should be organized in a way that a summary or interpretation is first shown before displaying detailed data. One participant mentioned that she could not give any suggestion about how to display the data or whether notifications are useful, as the app or its algorithm may not be trustable in the first place.

#### At-Home Examinations and Current Practice

Participants were asked whether they could imagine replacing a limited number of in-person obstetrician-gynecologist visits by conducting at-home measurements. One participant agreed with this idea but mentioned that it depended on the pregnancy and potential complications. One stated that fewer appointments could be convenient when a long drive to the doctor’s office is necessary. Another participant mentioned that she overall liked the appointments, but those where the physician was not involved could be potentially replaced. A total of 4 of 9 participants mentioned that they preferred the appointments, as they felt safer (1/4) and were able to ask questions (2/4). The question was not asked in 2 interviews as a result of the semistructured approach.

#### Data Donation: Sharing Data for Research Purposes

Lastly, data sharing for research purposes was discussed with participants. All but one woman stated they would share their data for research purposes, and 6 of 11 explicitly mentioned that the data should be anonymized. Several additional points were discussed by participants: security was perceived as important (1/11); the study and ethics committee should be named (1/11); participants would not share data if the data were invasive (1/11); a clear data protection policy is important (1/11); and data users must follow regulatory recommendations (1/11). One participant stated that she is privacy aware in everyday life but does not have these concerns regarding maternal record data.

## Discussion

This work aimed to assess usability and perceptions of a wearable-integrated DMR smartphone app. A user study was conducted for this purpose.

### Key Results

The app’s SUS score was a mean 79.09 (SD 18.38). This is in the 85th percentile compared to literature benchmarks and represents above average usability [33]. The app scored “above average” in all categories but “novelty.” We believe this is attributable to our close copy of the analogue (paper) version of the German maternity record (Mutterpass). It was not our aim to redesign the document, as this is outside the scope of this work. Therefore, we closely copied the document in the maternity record section of our app but did not explore all benefits for the health care setting of a digital redesign.

TCTs in all tasks were higher than those modeled with GOMS, with the highest differences in tasks 1, 2, and 8. The long time taken for task 2 can be attributed to the atypical app installation process. As the app is a PWA, it is installed by using browser-delivered functionality as opposed to an app store. The respective instructions are lengthy and require participant effort. In task 8, users were able to choose different visualization options through a “slide-to-the-side” bar on top of the page. This functionality and the choice of visualization options was not obvious to users. The visualization options were presented using a “slide-to-the-right” bar, also resulting in user experience problems. Users performed think-alouds throughout the tasks. This may also have had a negative effect on task completion time.

Most errors occurred in task 2 (ie, app installation, during which 17 errors occurred; Table 2). The menu button was placed on the top left of the page. It was thus not visible unless users scrolled to the top of the page, which resulted in corresponding errors. Task 1 (user creation and login; 2/11, 18%) and task 8 (find heart rate value; 5/11, 46%) had low task success rates. In the first task, users tried to log in without having previously registered an account, which can be ascribed to poor study instructions. Task 8 suffered from the presentation of visualization options, as explained in the previous paragraph. The study was overall very helpful in finding usability problems in our app, which we continue to improve for future use. This equally applies to the feature requests made by participants.

The trustworthiness of pregnancy apps was considered as important by several women, which favored apps from official sources. This underlines our overall concept.

Interviews provided insights into the perception of individual aspects of our DMR concept. Opinions regarding the overall concept of a wearable-integrated DMR app were mixed. Participants stated that a clear benefit must be communicated, and this benefit should be communicated by the caretaking obstetrician-gynecologist. Participants that experienced pregnancy complications in the past were overall more open toward the idea.

### Comparison With Prior Work

To the best of our knowledge, previous work, including think-aloud usability sessions or studies focusing on wearable integration in maternity care, is limited to date. Our work is the first to examine usability in a DMR environment using the think-aloud method and the first overall to investigate aspects of usability when integrating wearable data into a DMR app.

Usability is often reported as a secondary element or through postuse surveys or questionnaires [21,22,34]. Scott et al [35] analysed commercial pregnancy apps and found that only 2 of 10 apps had full usability. An online tool for gestational diabetes reached a SUS score of 70.9 [36]. The only study we identified that used think-aloud sessions investigated an app for pregnancy-related work advice [37]. A total of 82 usability problems were identified, and the overall mean SUS score was 68. In light of these findings from the literature, we believe our app (mean SUS score 79.09, SD 18.38) overall has high usability compared to its peers. It furthermore benefited and will continue to benefit from our UCD approach.

Our results from the semistructured interviews are overall in line with previous work. Groenen et al [38] found that users do not start a DMR if they feel it has no perceived value and that physicians play a key role in the adoption of technology. In this previous work, participants similarly stated that pregnancy complications increase the value of technology. Other work confirms these findings [15,16].

### Strengths, Weaknesses, and Contribution

Several limitations apply to our work. Participants did not conduct the study on their phones, but instead used a phone interface on their desktop computers. This was necessary due to COVID-19–related restrictions. Usability problems related to the actual end device and its use (eg, smartphone or tablet) cannot be discovered in such a setting. The SUS score was shown as a questionnaire in the app. We used a vertical instead of a horizontal layout for the Likert questions, which could influence results. Overall, participants had a both a comparably high education level as well as household income. Women with low digital affinity may be less likely to participate in a study such as ours, and could thus be underrepresented. Interviews may have been biased in the sense that not every participant was asked every question, as they were designed as semistructured sessions to catch overall perceptions and impressions. The semistructured interviews conducted as part of our study design could benefit from additional participants. We did not use a fixed threshold since the last pregnancy as an inclusion criterion. Thus, the time since the last pregnancy varied between participants. This may have influenced participants’ responses.

Our study combines several strengths: Regarding the usability study and think-alouds, a sufficient number of women with previous pregnancy experience participated, and the participant size was adequate for the proposed study design [39,40]. We are the first to explicitly conduct an observational usability study of pregnancy mobile health, compared to previous work that relied on postuse questionnaires. Furthermore, we are the first to evaluate several mobile health and ubiquitous health concepts, including the use of wearables in care practice, in one single prototype. We believe this descriptive prototype makes it easier for participants to understand the underlying concepts and the potential impact on their personal life. Additionally, we evaluated several connected topics of high importance in the realm of mobile and ubiquitous health in maternal care, particularly regarding information research, health anxiety, reaction to potential alerts, and data sharing for research.

### Future Work and Areas for Innovation

We propose several areas for future work. These proposals are based on statements of the participants, the literature, experiences during app development, and lessons learned from conduct of this study. They target both industry innovation and future scientific work.

Participants stated that they would appreciate additional explanations within the DMR section of the app. The existing German paper-based hand-held maternity record largely relies on medical terms and abbreviations. Based on the interviews and our clinical experience, these are not understood by many women. The DMR has the potential to provide understandable additional information in close proximity to the respective fields. This can include easy-to-understand explanations and expert videos; it could even be tailored to individual measurement values or findings.

The visualization and communication of recorded and potentially automatically assessed wearable data must be considered in future work. There was no consensus on whether data should be completely accessible by or hidden from the user. Some participants were aware of potential risks such as cyberchondria and preferred not to view the full data in the first place.

An important related question is the reaction to abnormal measurements. Should the user or the physician be notified? Participants were equally split regarding this question. One way to address these mixed opinions could be a user-controlled setting.

DMRs have the potential to alleviate media discontinuties [23]. They can improve information flow from women to caregivers as well as between caregivers. Thus, DMRs may be a tool to improve overall care.

Recommendations for the use and application of wearables in routine care may be helpful for both pregnant women and caregivers. An important question for future discussion is the measurement reliability of devices and interpretation of the generated data. The latter is relevant as data are generated “in the field” outside the supervision of medical professionals and could be of lower quality and reliability.

Sharing data for research was favored by most participants, although this finding may be biased given that we only interviewed participants that decided to take part in a scientific study in the first place.

Our work only investigated usability, perception, and opinions on several related topics among women who had been pregnant in the past. Longitudinal studies during pregnancy may be helpful to examine how app interaction, opinions, and related phenomena (such as cyberchondria) change throughout pregnancy.

### Conclusion

We were the first to assess the concept of a wearable-integrated DMR app in terms of usability and overall perception. The app combined several concepts: the maternity record itself, information sections, selected consumer pregnancy app functionalities, and the integration of wearable data. Semistructured interviews on online information research, the use of wearable devices, integration into routine care, and data sharing for research completed the study.

Our work found good overall usability and was helpful in identifying usability problems as well as errors. Participants were of mixed opinions regarding the integration of wearable technology into prenatal care. Clear benefits of such devices must be communicated to prospective users to ensure user acceptance. Pregnant women rely on the opinion and guidance of their caretakers, particularly gynecologists and midwives, who thus play a key role in the adoption of mobile and ubiquitous health technology such as wearables in maternal care.

Opinions on the display of wearable data, its evaluation, alert levels, and handling of potential alerts were mixed and highly individual. Some women preferred to see all data, while others explicitly did not want access to it. Future work should thus investigate different personalized options for wearable data display, interpretation, automated evaluation, and potential reaction to alerts.

## Supplementary material

10.2196/50765 Multimedia Appendix 1 Retrospectively translated English version of example screenshots of the developed app (from top left to bottom right): “Start,” “My tasks,” “MAPI questionnaire,” “Installation guide,” “My measurements,” “Main menu,” “Ultrasound section,” and “Information” section. The app is intended for a German audience and was thus originally developed in German. This version was not used in the study but is provided for reference and clearer presentation.

10.2196/50765 Multimedia Appendix 2 Example app walkthrough based on the study procedure.

10.2196/50765 Multimedia Appendix 3 Tabular overview of participants’ responses during the semistructured interviews.

## Abbreviations

DMR: digital maternity record
GOMS: goal, operators, methods, and selection rules
PWA: progressive web app
SUS: System Usability Scale
TCT: task completion time
UCD: user-centered design
UEQ: User Experience Questionnaire
