# Supplementary figures and images for: Usability and Perception of a Wearable-Integrated Digital Maternity Record App in Germany: User Study
Source: JMIR Pediatr Parent. 2023 Dec 15;6:e50765. doi: 10.2196/50765 (PMC10750977; doi:10.2196/50765)

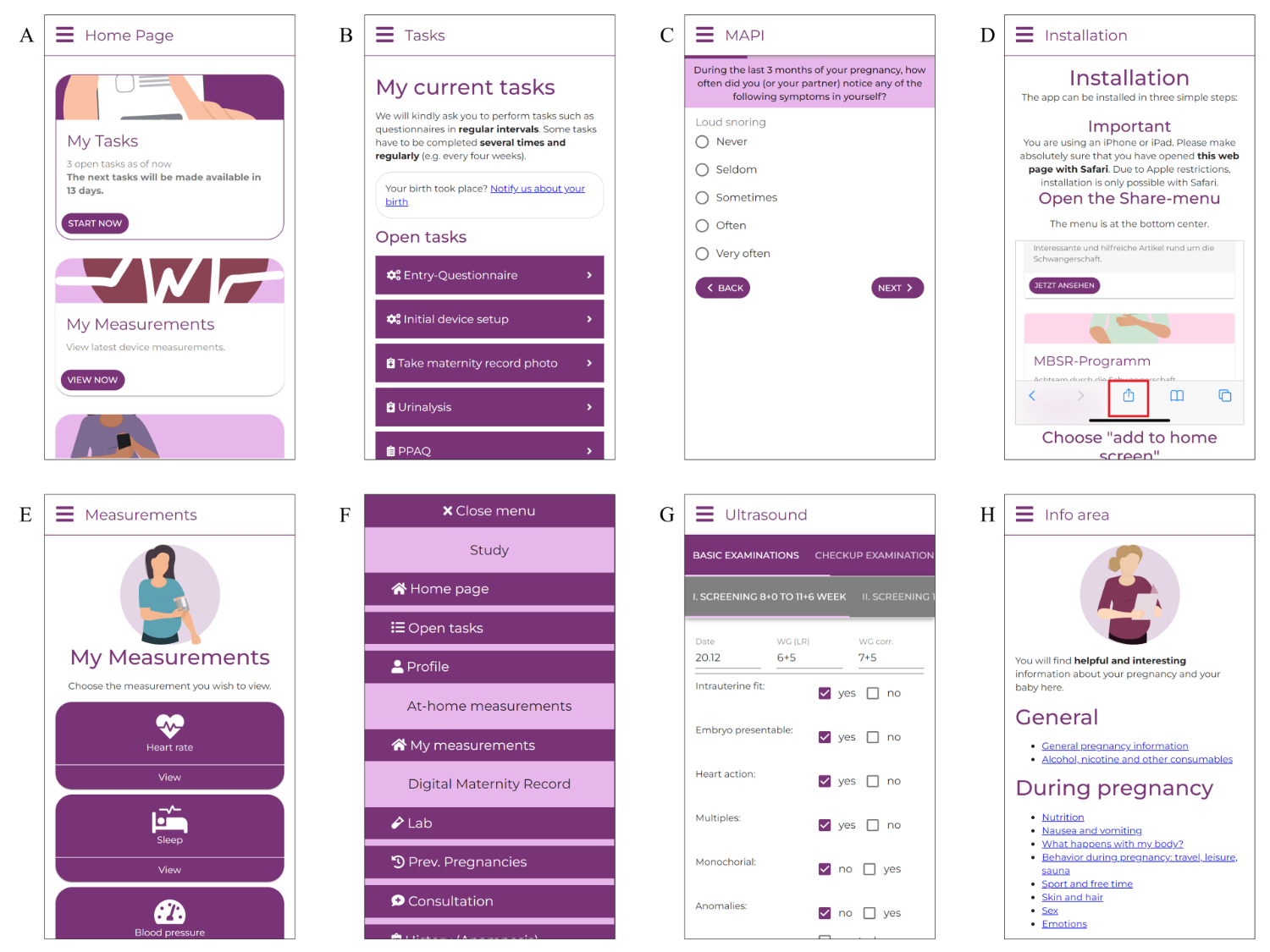

Supplement: Multimedia Appendix 1 [file pediatrics-v6-e50765-s001.png]
