# Supplementary material for: Usability and Perception of a Wearable-Integrated Digital Maternity Record App in Germany: User Study
Source: JMIR Pediatr Parent. 2023 Dec 15;6:e50765. doi: 10.2196/50765 (PMC10750977; doi:10.2196/50765)
Supplement: Multimedia Appendix 2 [file pediatrics-v6-e50765-s002.pdf]

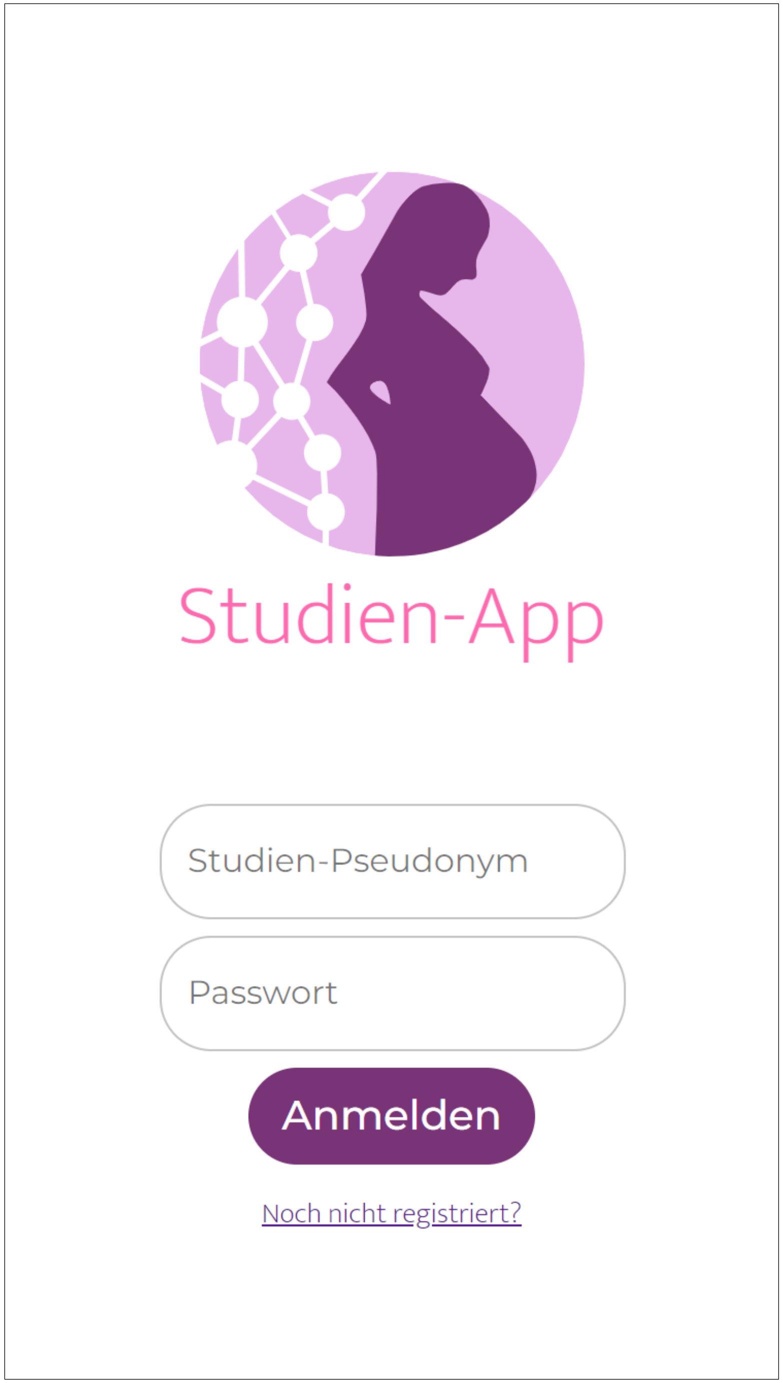

Task 1:
Create a new user and login

App start page

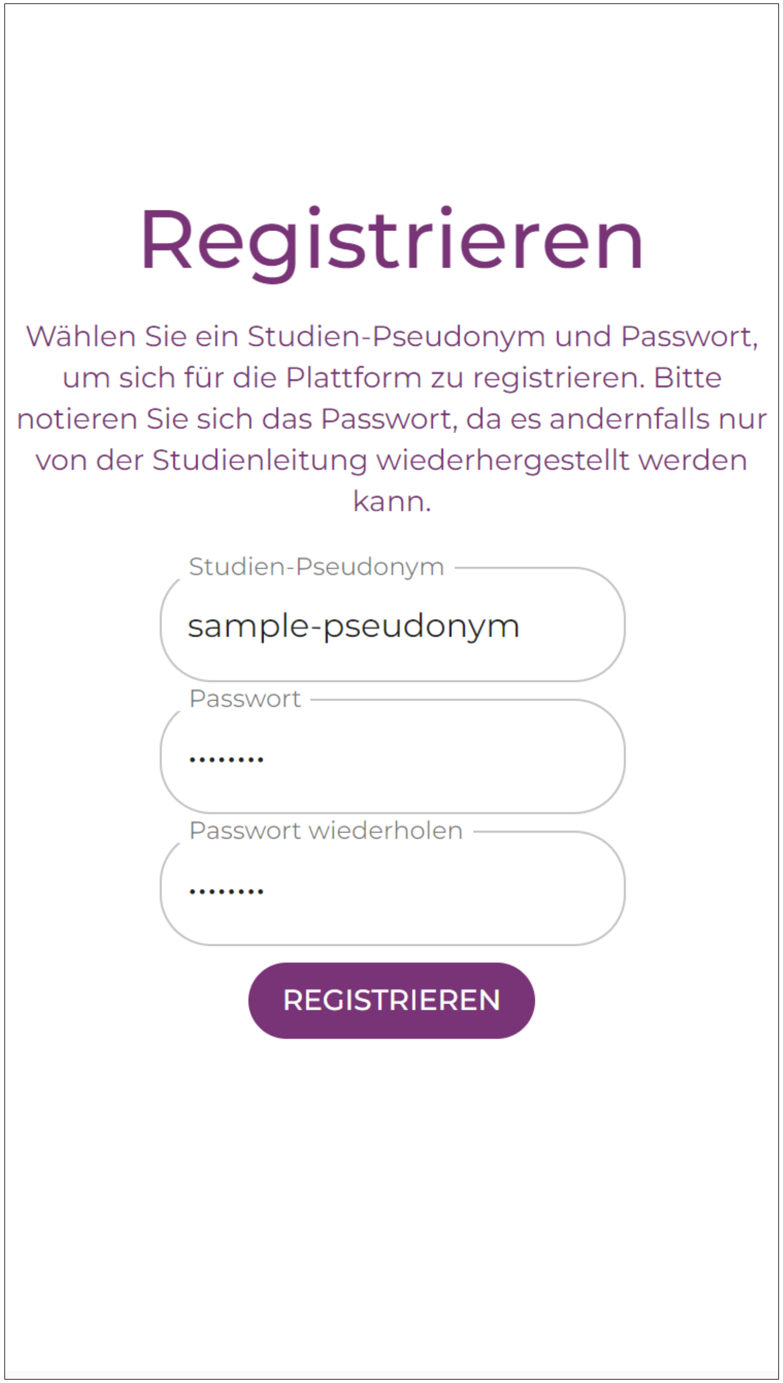

Task 1:
Create a new user and login

Registration page

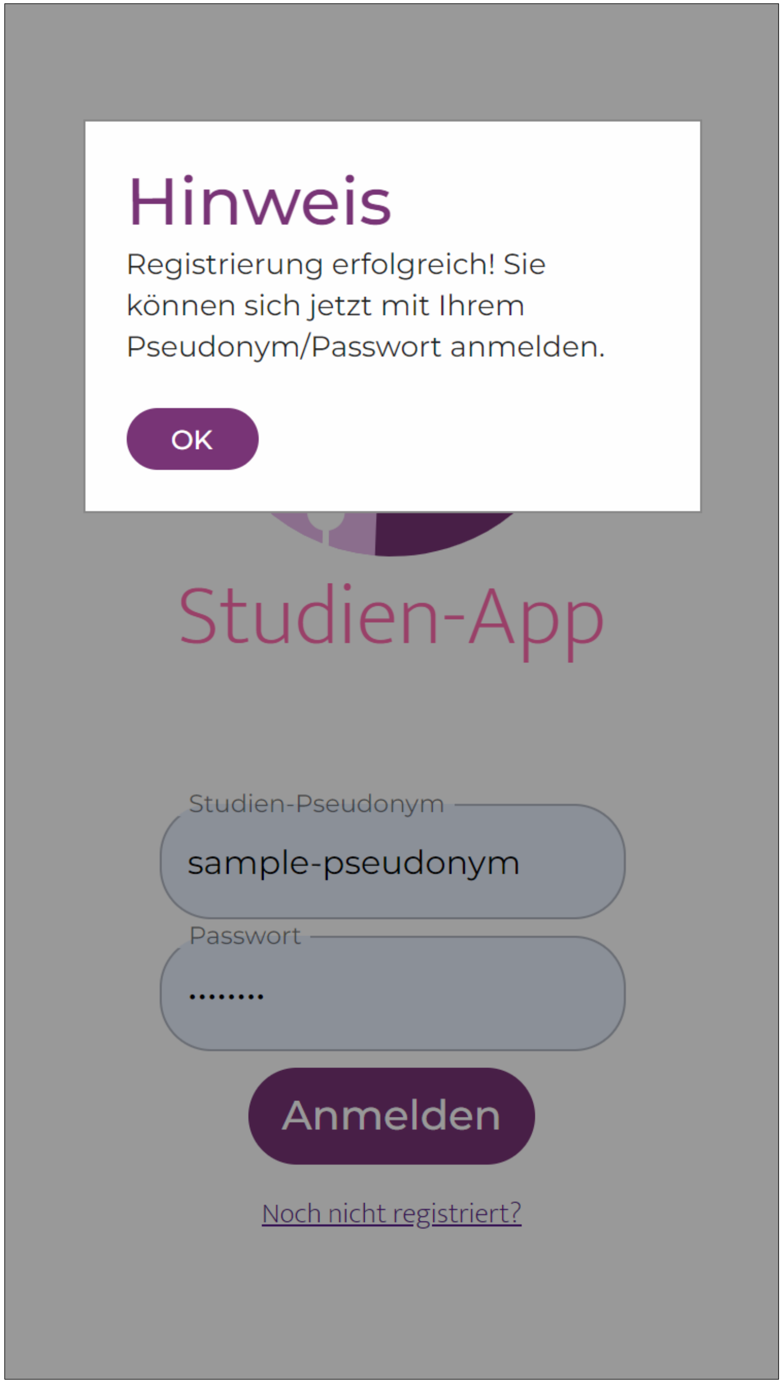

Task 1:
Create a new user and login

Registration successful message

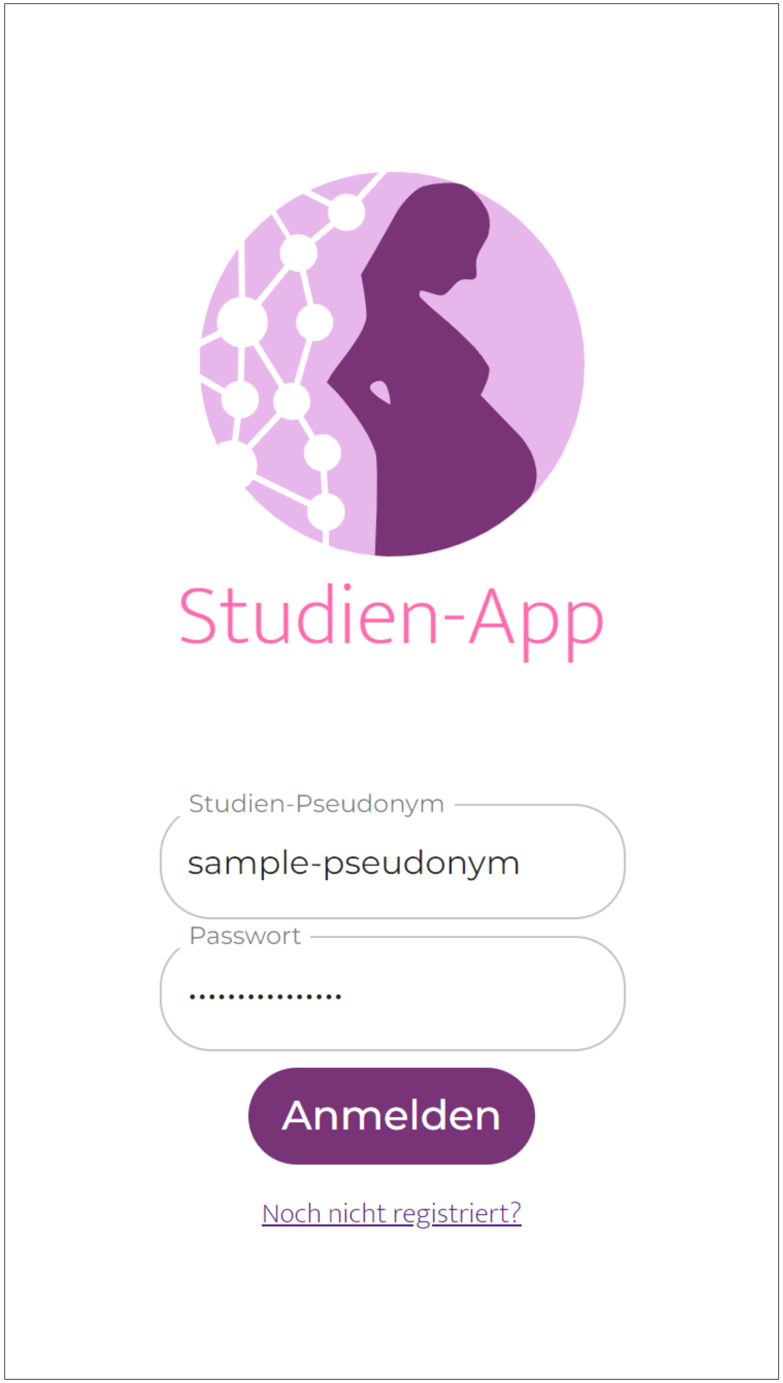

Task 1:
Create a new user and login

User login page

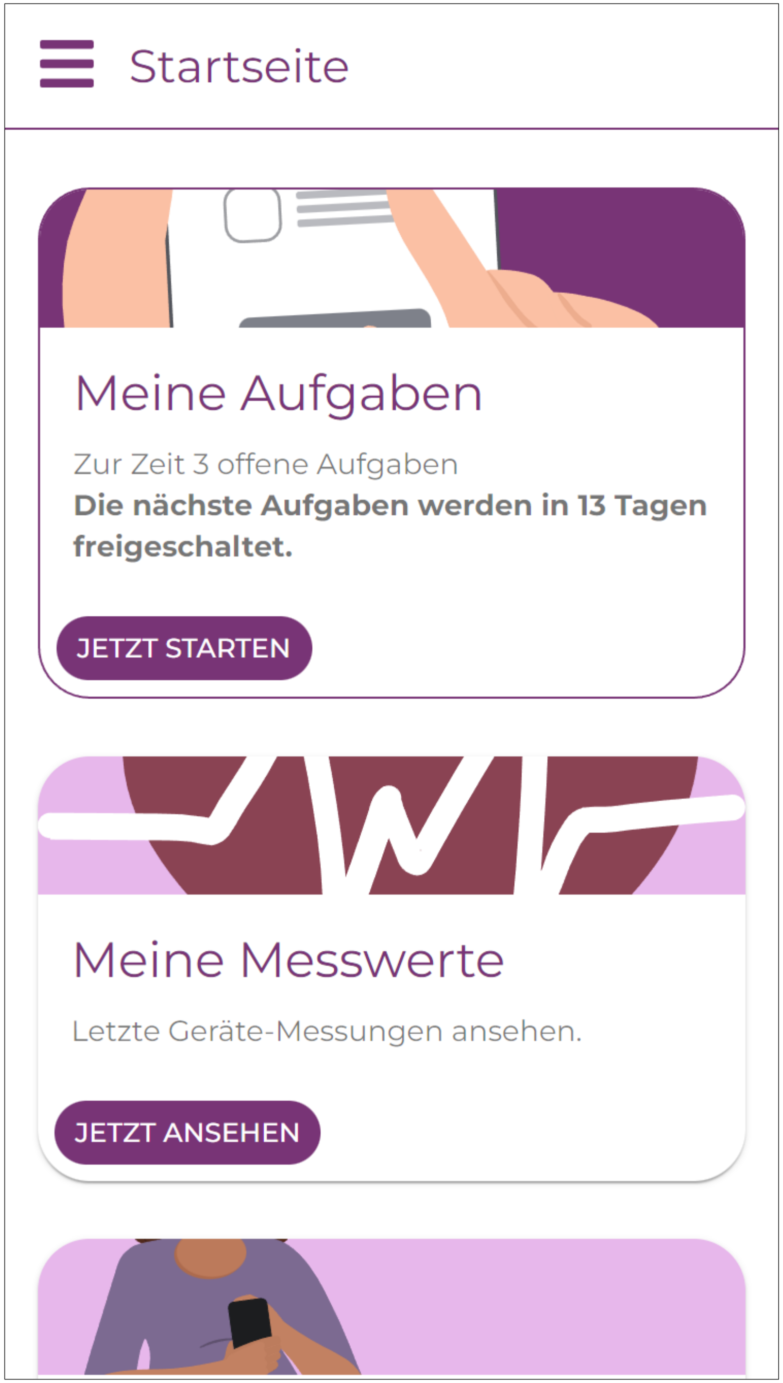

Task 2:
Install the app on the mobile phone

App start/overview page

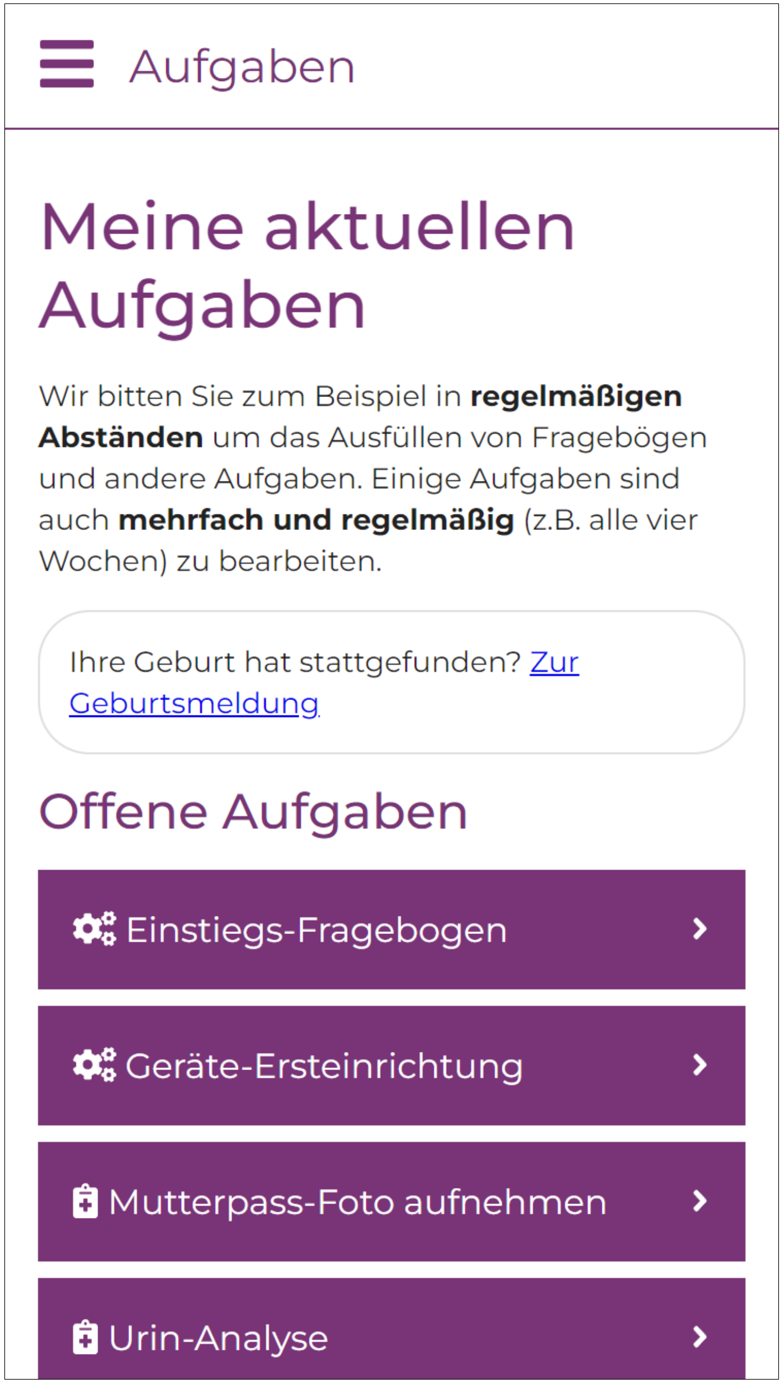

Task 2:
Install the app on the mobile phone

“My tasks“ list

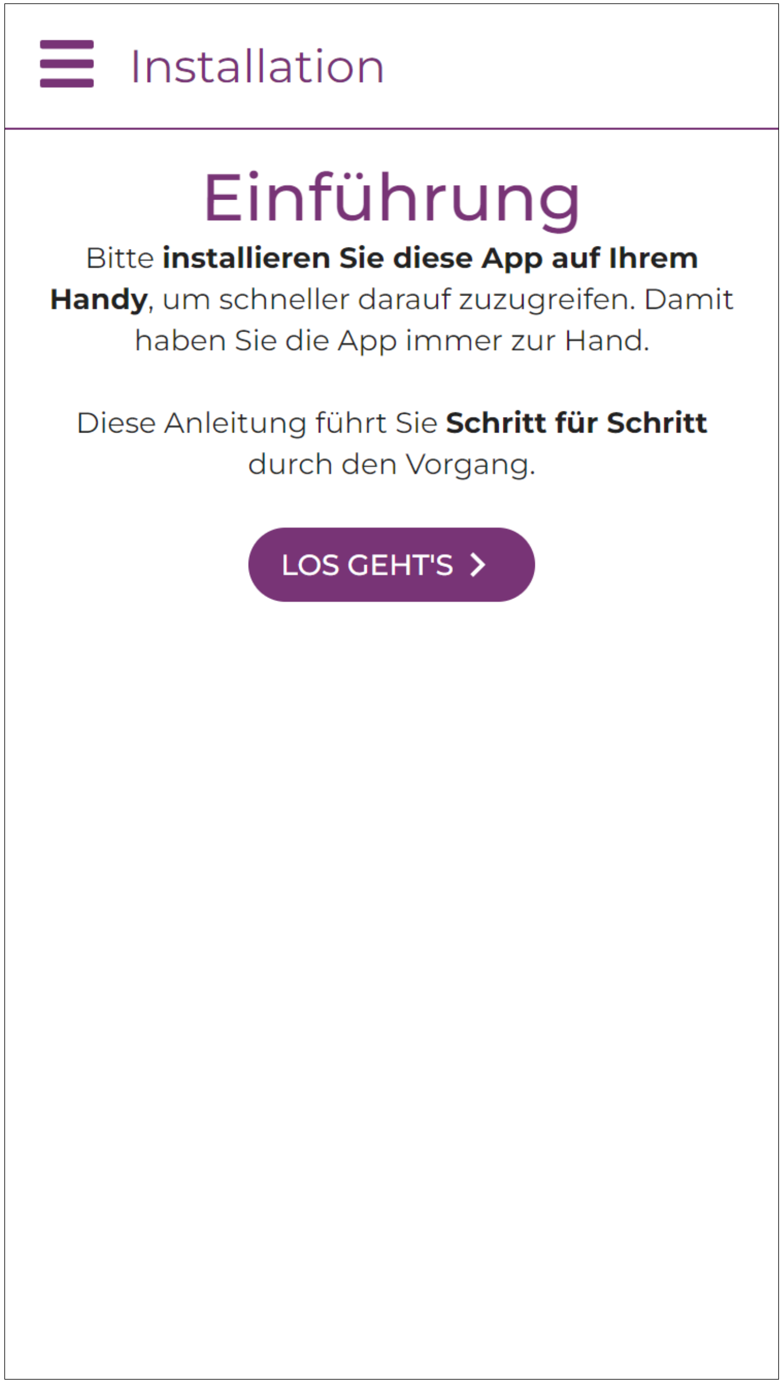

Task 2:
Install the app on the mobile phone

Installation instructions (start)

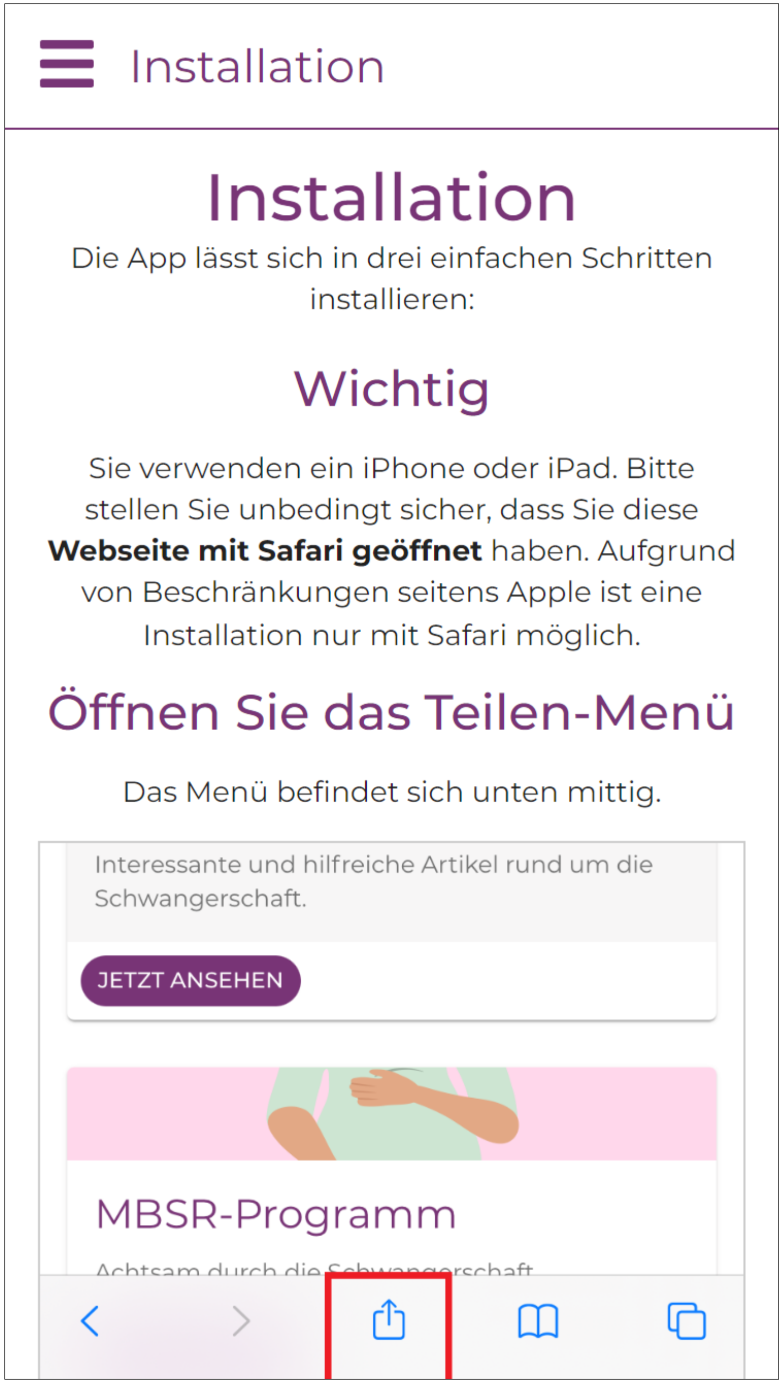

Task 2:
Install the app on the mobile phone

Installation instructions

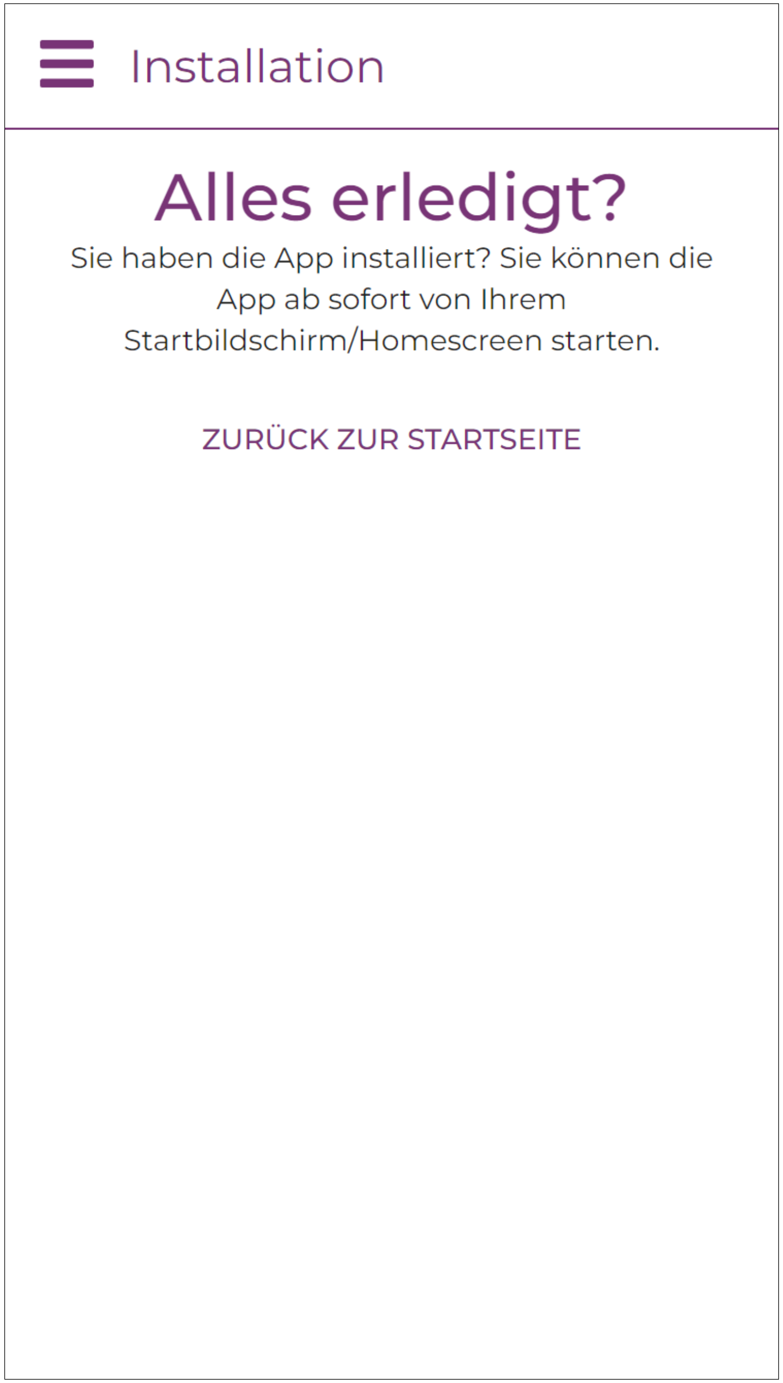

Task 2:
Install the app on the mobile phone

Installation confirmation page

Task 3:
Free exploration

Not shown in this document

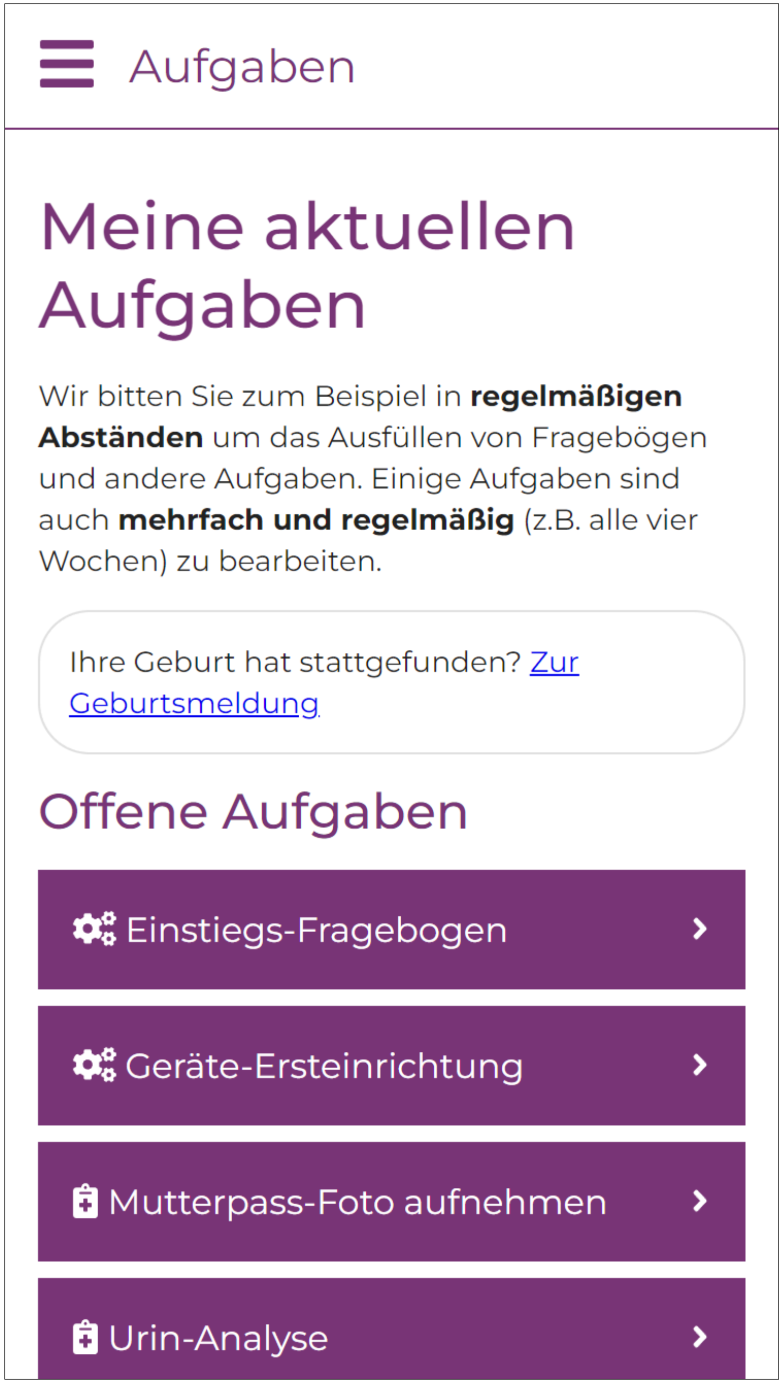

Task 4:
Answer Multivariable Apnea Prediction Index Questionnaire

“My tasks“ list

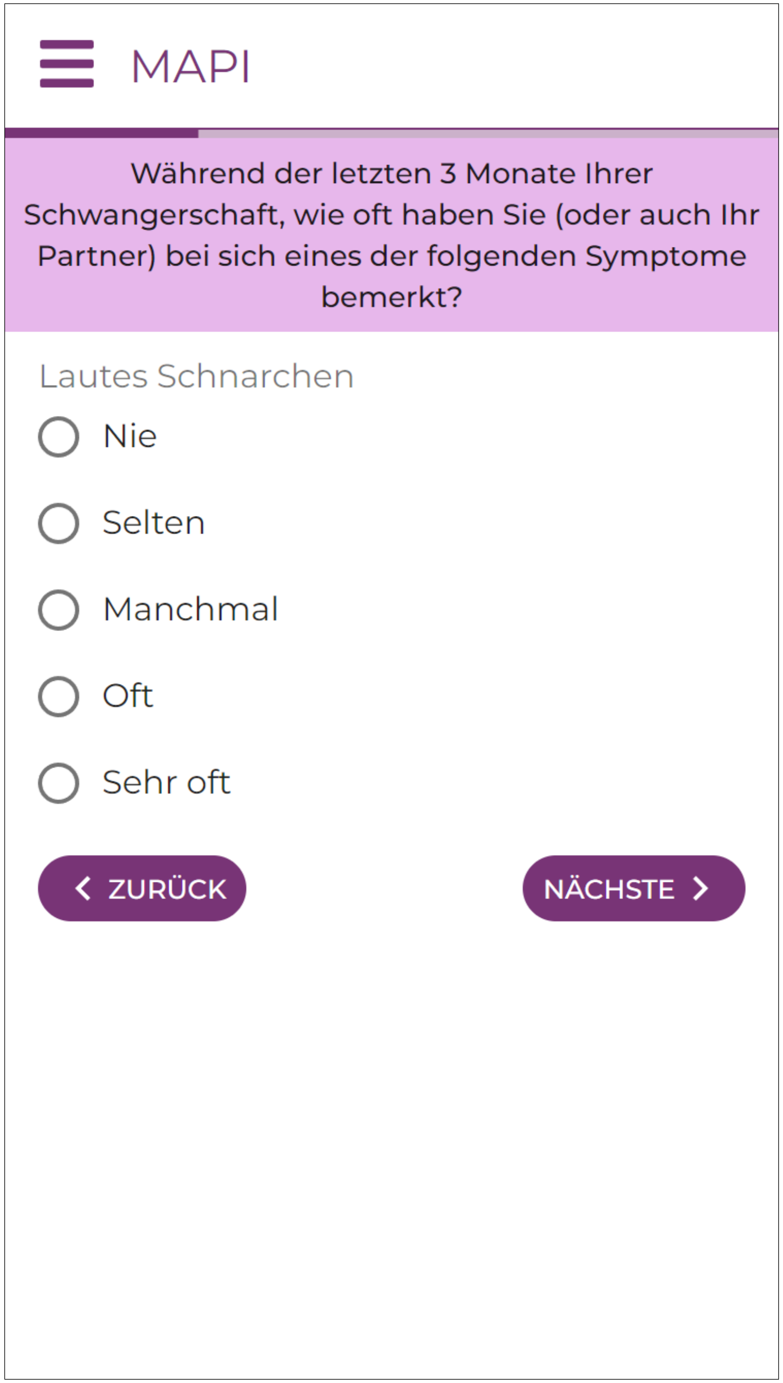

Task 4:
Answer Multivariable Apnea Prediction Index Questionnaire

Exemplary question of the multi-question questionnaire

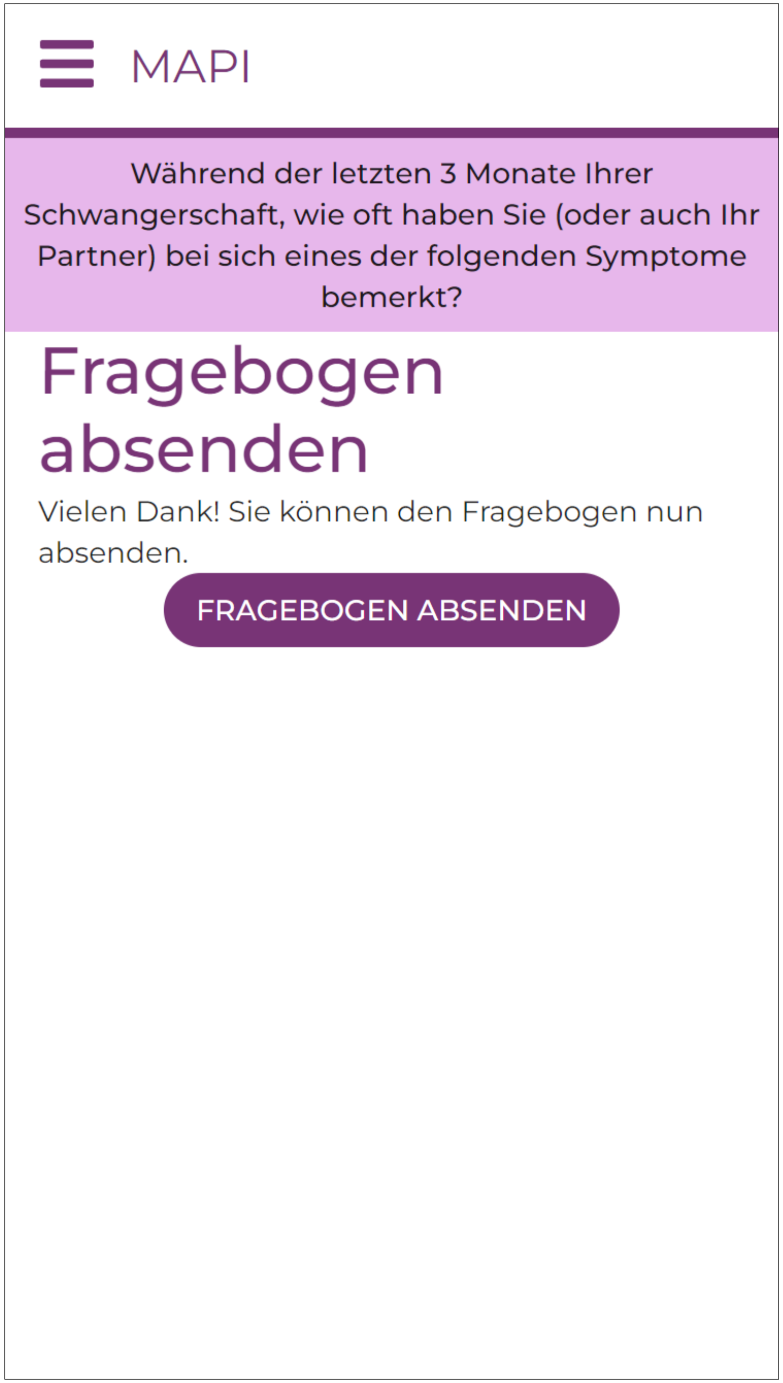

Task 4:
Answer Multivariable Apnea Prediction Index Questionnaire

Finish questionnaire page

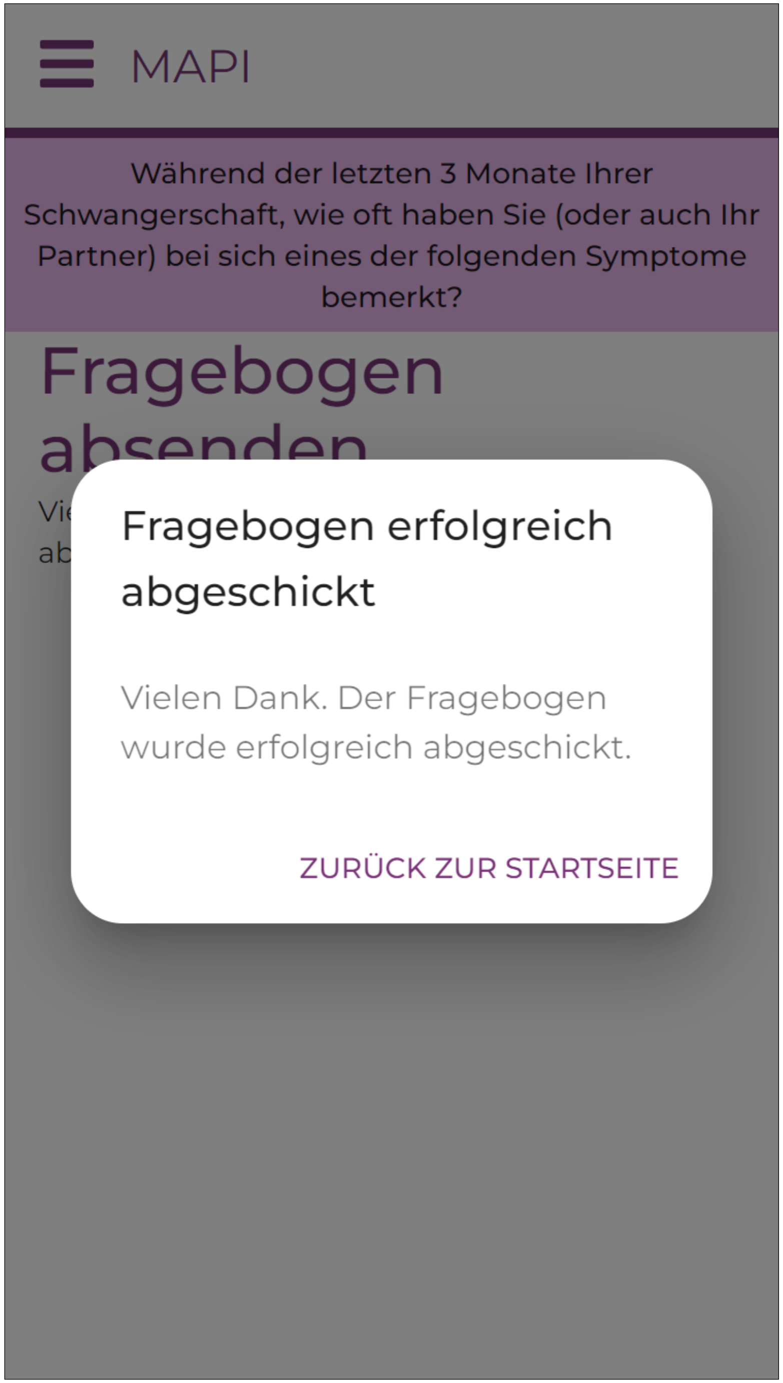

Task 4:
Answer Multivariable Apnea Prediction Index Questionnaire

Questionnaire successfully sent page

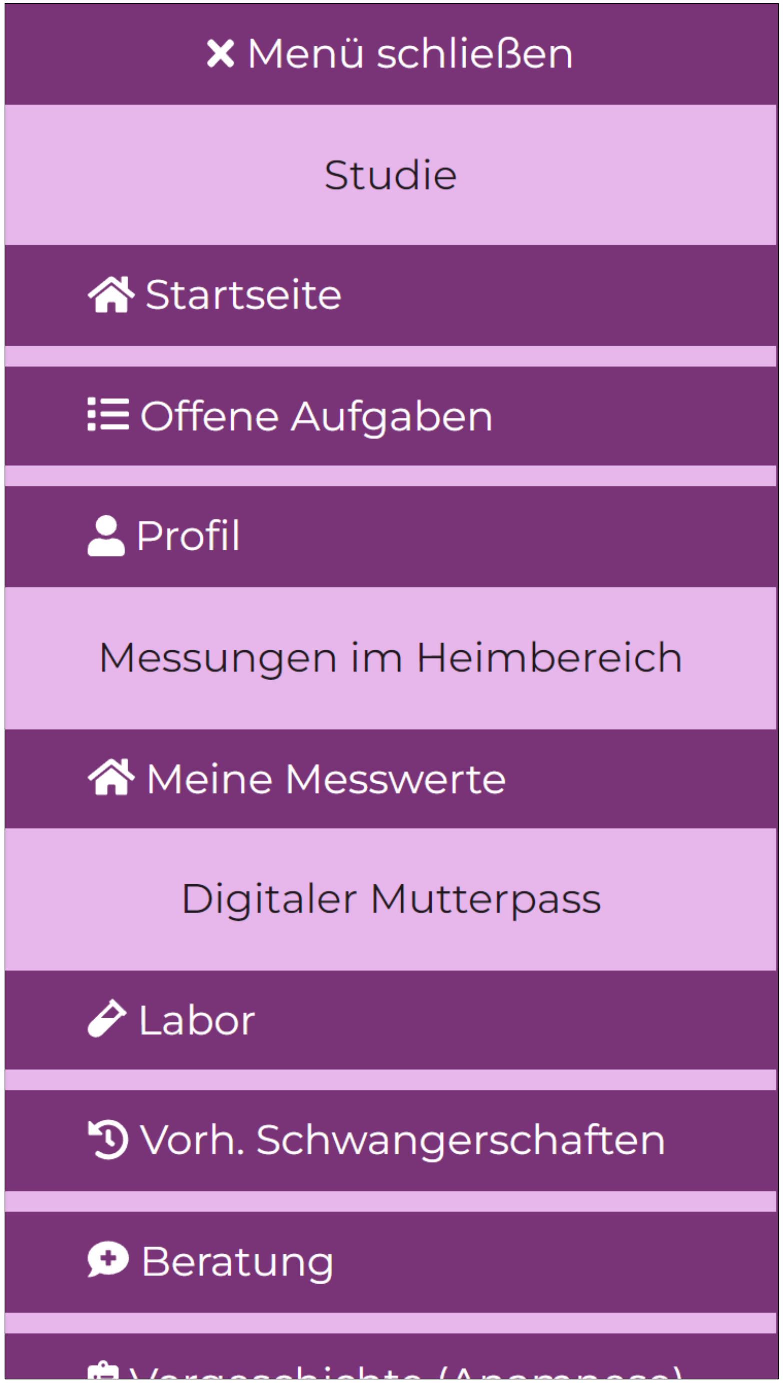

Task 4:
Explore ultrasound section

Main app menu, ultrasound section item below the fold

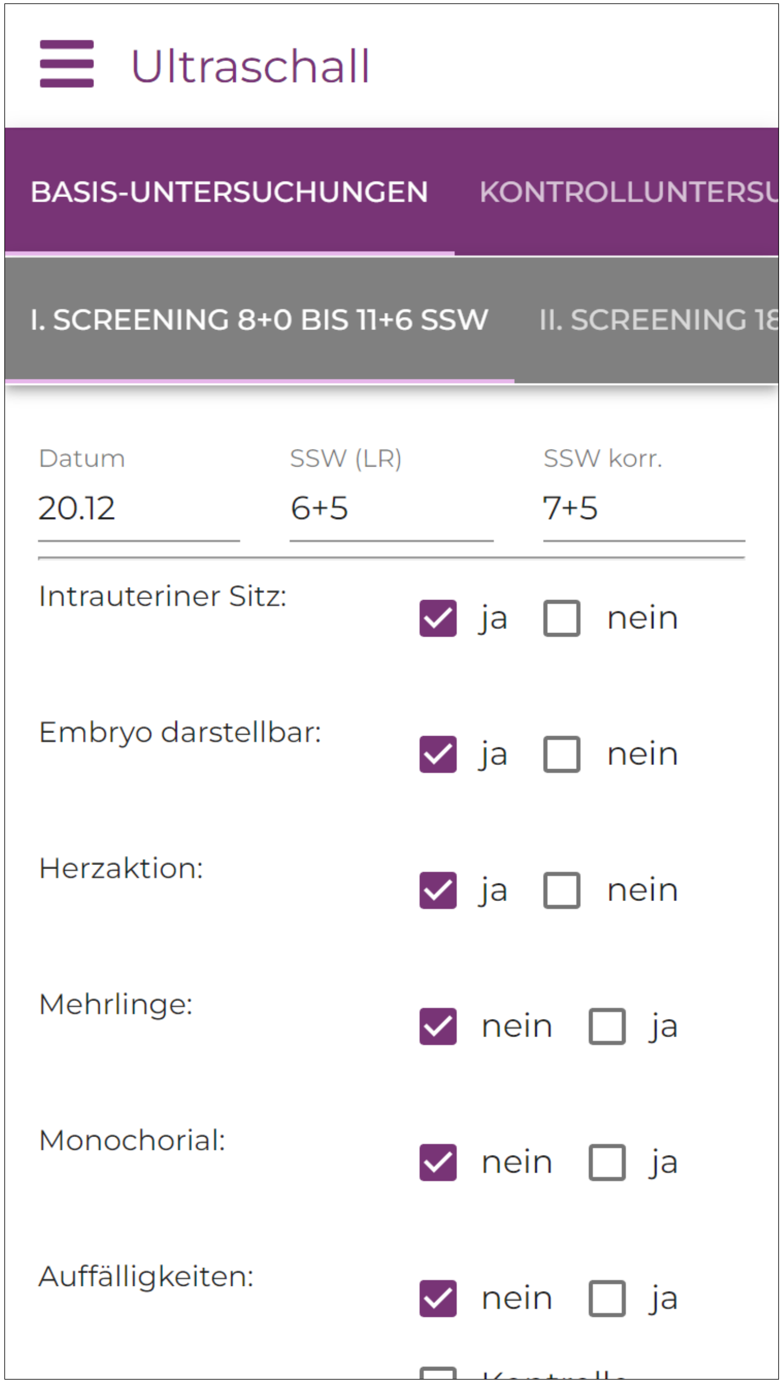

Task 5:
Explore ultrasound section

First screening page

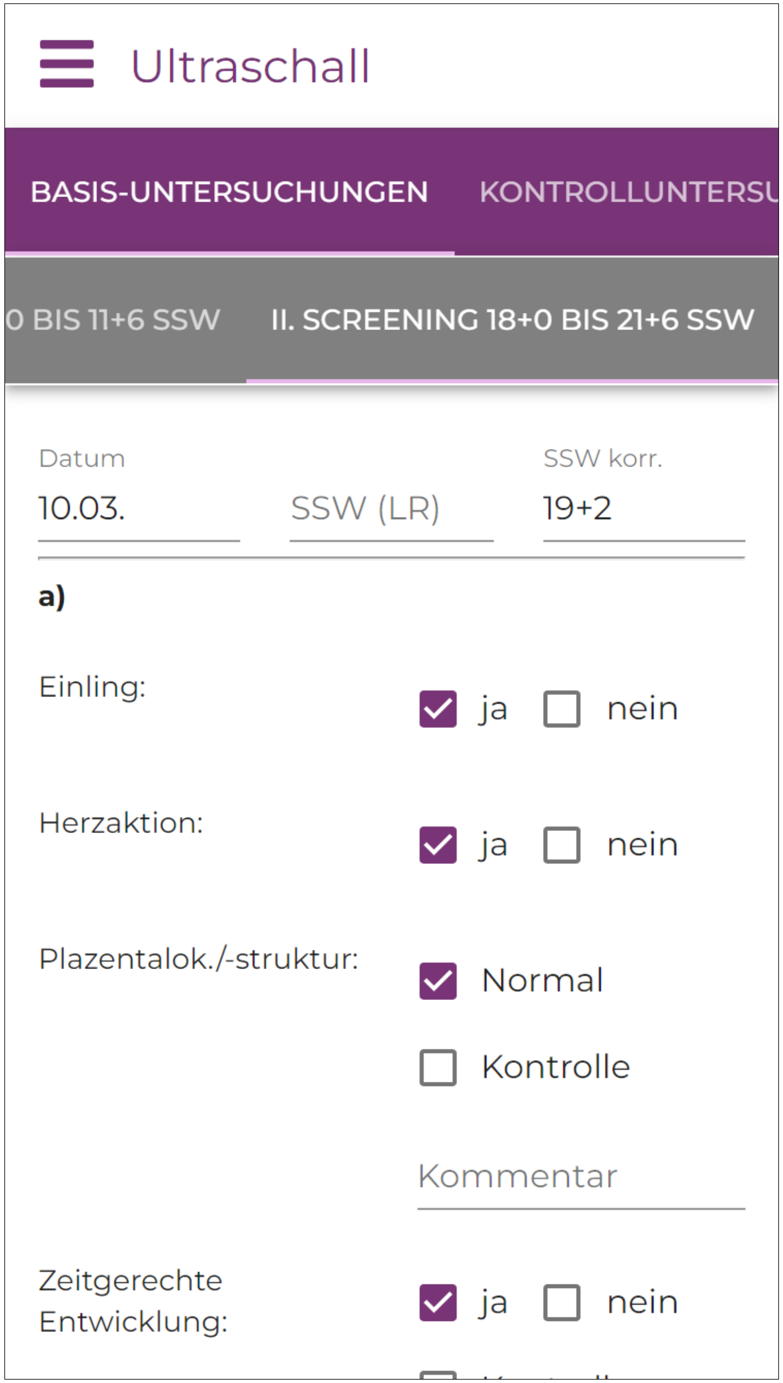

Task 6:
Find remarks of second ultrasound checkup

Second screening page

Task 7:
Explore visualization options

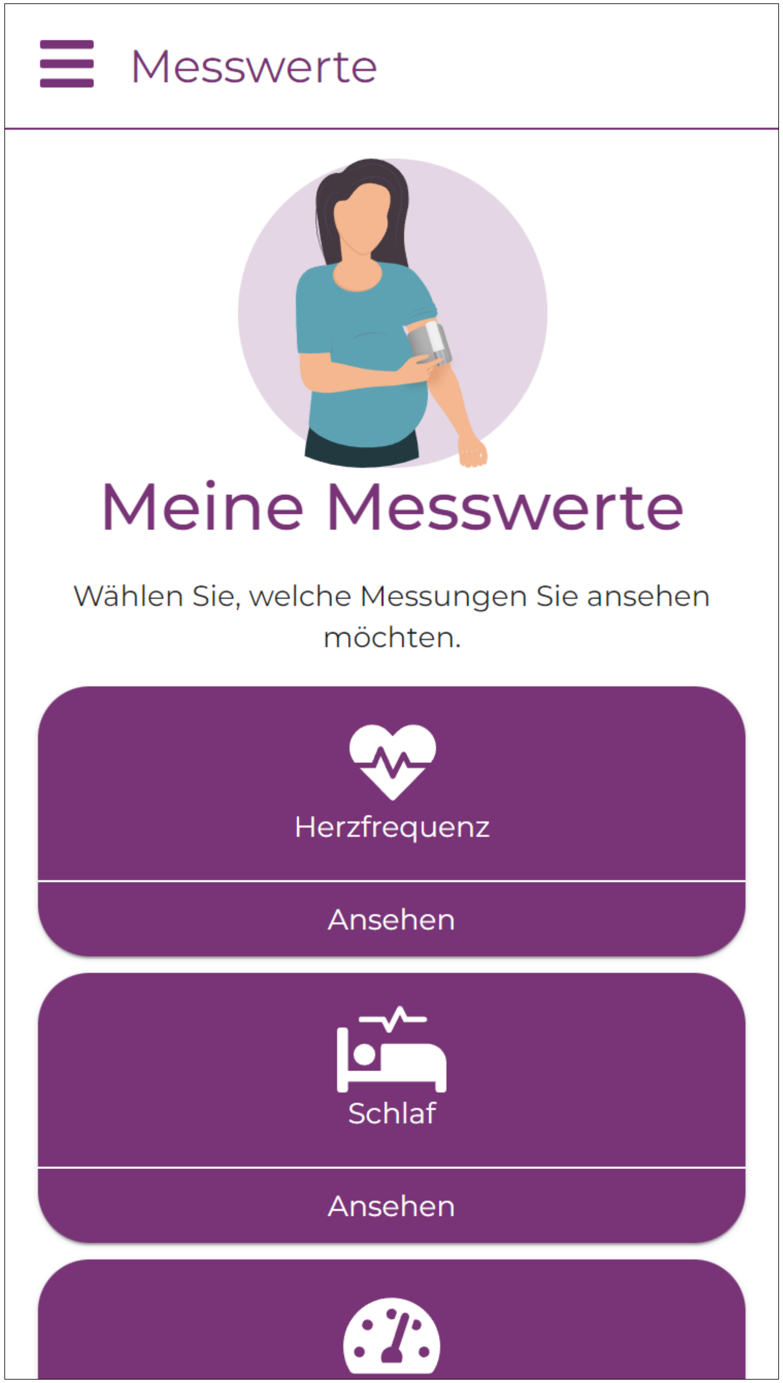

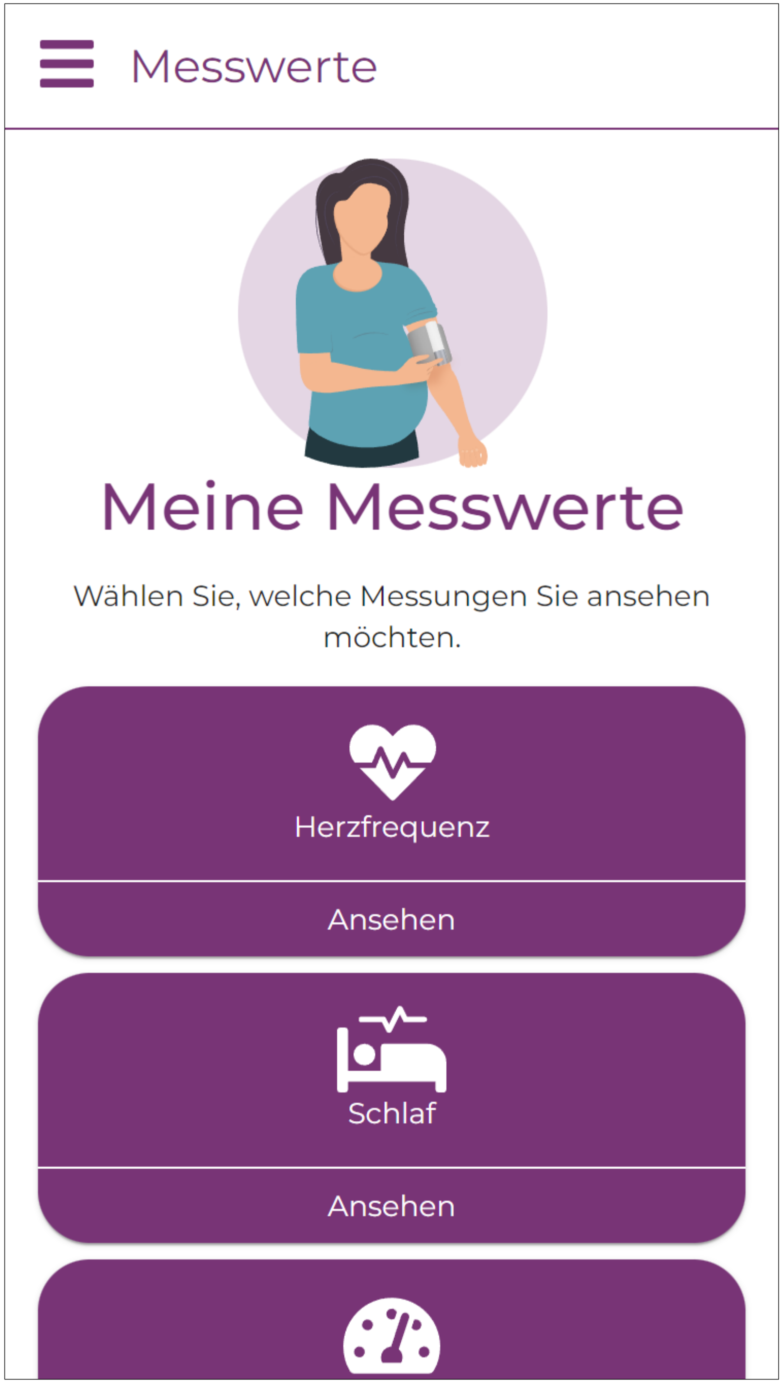

Task 8:
Find a heart rate value

Info-Bereich

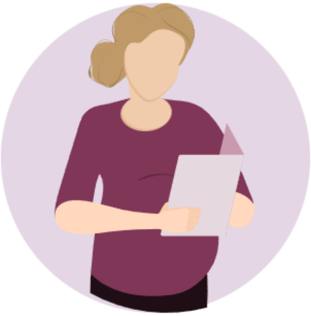

Hier finden Sie **nützliche und interessante Informationen** rund um Ihre Schwangerschaft und Ihr Baby.

Allgemein

- Allgemeines zur Schwangerschaft
- Alkohol, Nikotin und sonstige Konsumgüter

Während der Schwangerschaft

- Ernährung
- Übelkeit und Erbrechen
- Was passiert mit meinem Körper?
- Verhalten in der Schwangerschaft: Reisen, Freizeit, Sauna
- Sport und Freizeit
- Haut und Haar

Task 9:
Find depression information

„Knowledge“ section overview page

Emotionen in der Schwa...

Informationen > Während der Schwangerschaft > Emotionen in der Schwangerschaft

Emotionen in der Schwangerschaft

‹ ZURÜCK ZUR STARTSEITE

Die Schwangerschaft ist eine Achterbahnfahrt. Hier wollen wir uns mit den Hochs und Tiefs in der Schwangerschaft und der Zeit danach beschäftigen.

Emotionen in der Schwangerschaft sind vielfältig. Vielleicht haben Sie ja auch schon festgestellt, dass Ihre Gefühle manchmal Achterbahn fahren.

Häufig sind Sie sehr glücklich über die Schwangerschaft und die Entwicklung ihres Kindes. Während der Schwangerschaft kann es aber auch Phasen geben, in denen es Ihnen nicht so gut geht und Sie trauriger sind als sonst.

Depression und Traurigkeit in der [illegible]

Task 9: Find depression information

„Emotions during pregnancy“ article

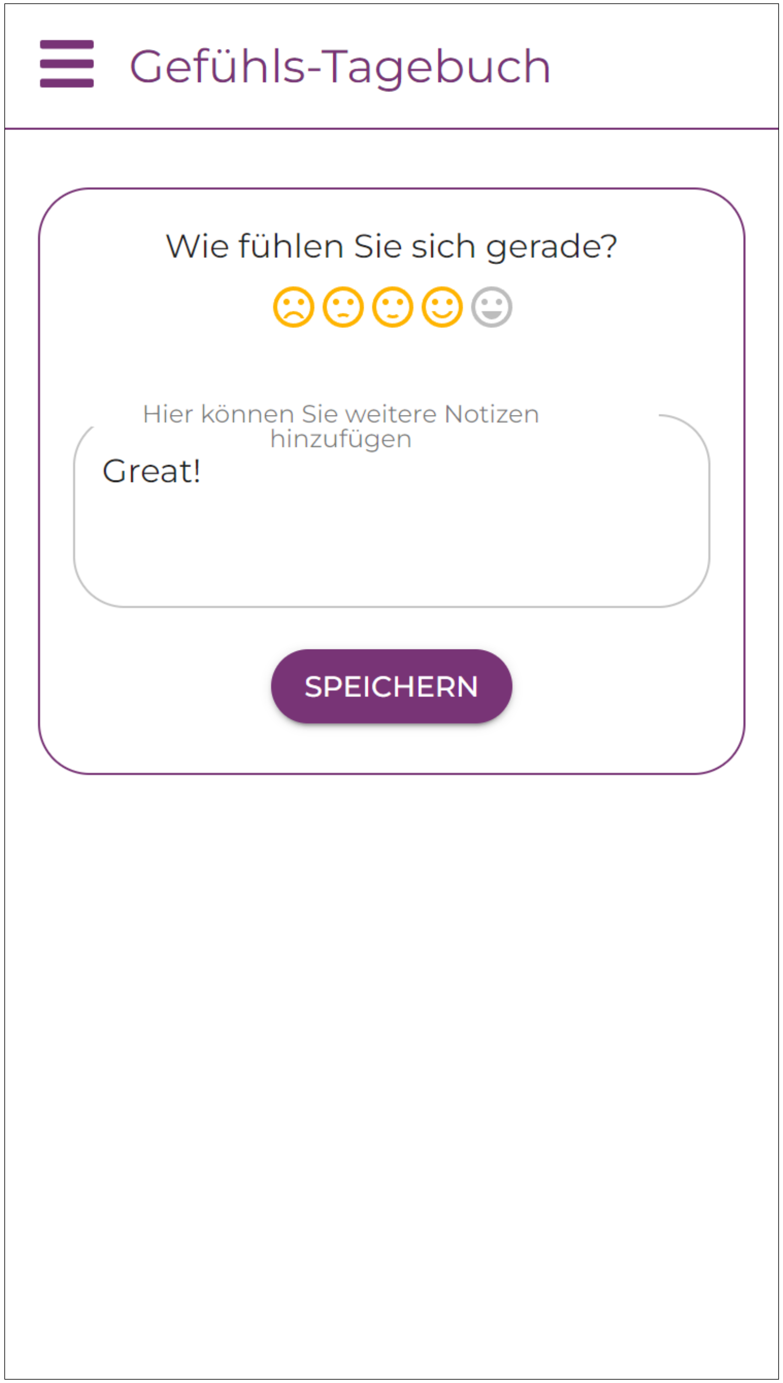

Task 10:
Enter information in mood journal

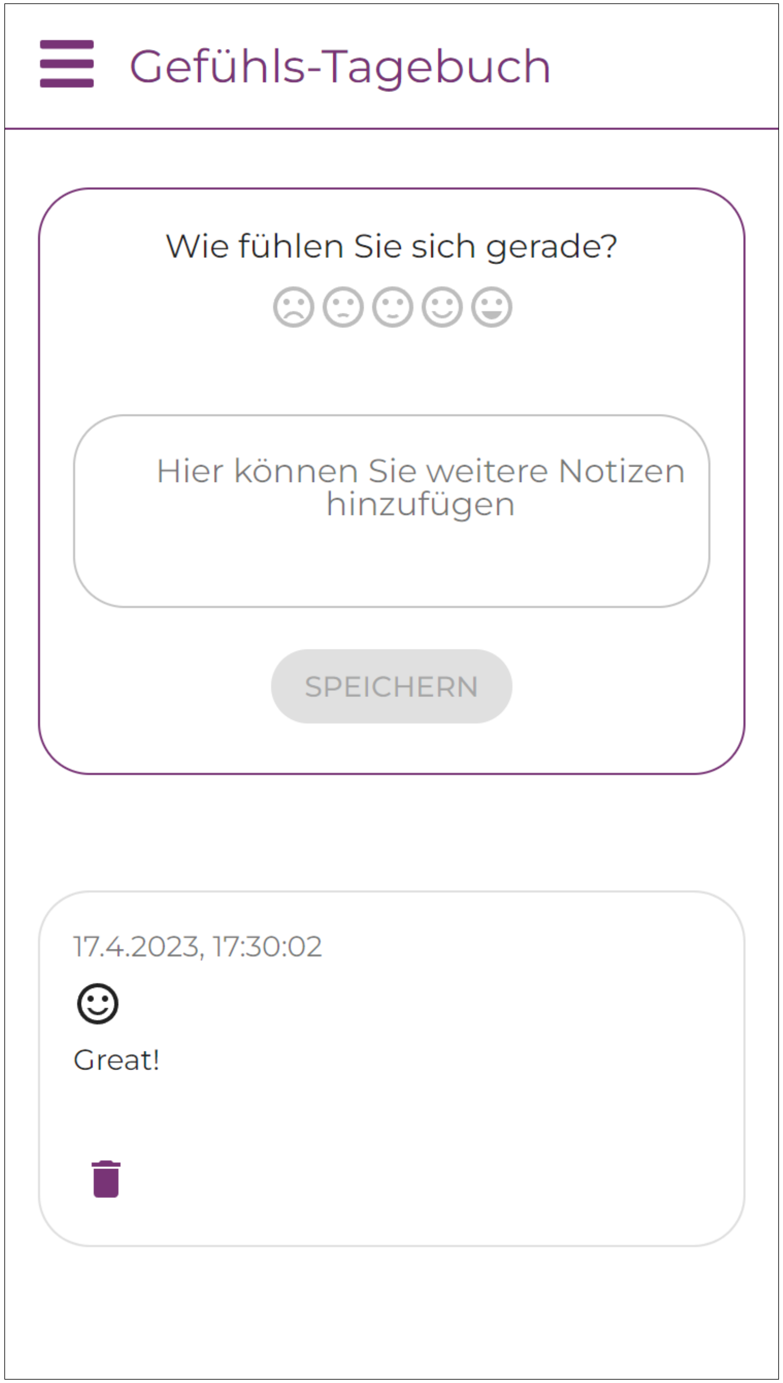

Task 10:
Enter information in mood journal

Page after submission of an item

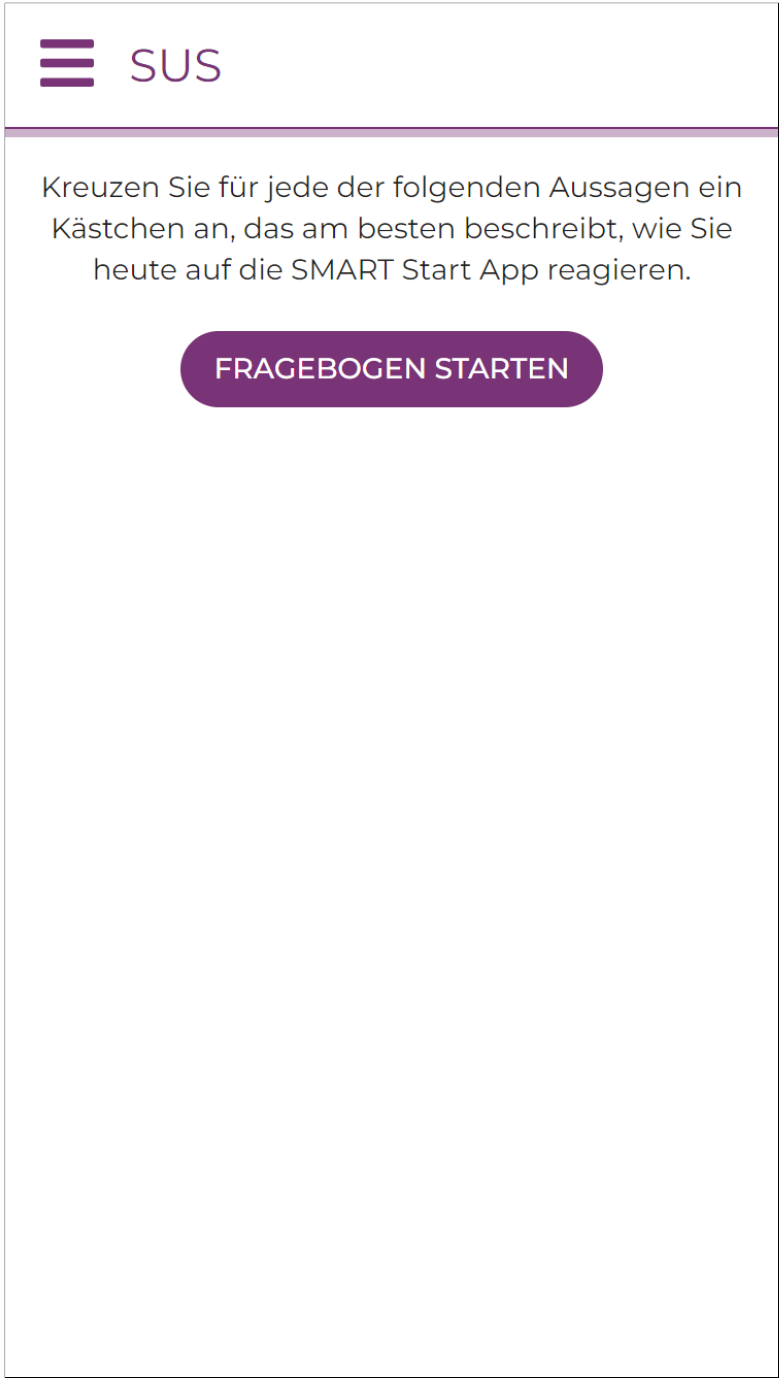

Questionnaires: SUS

Introductory screen

Questionnaires:
SUS

Exemplary question

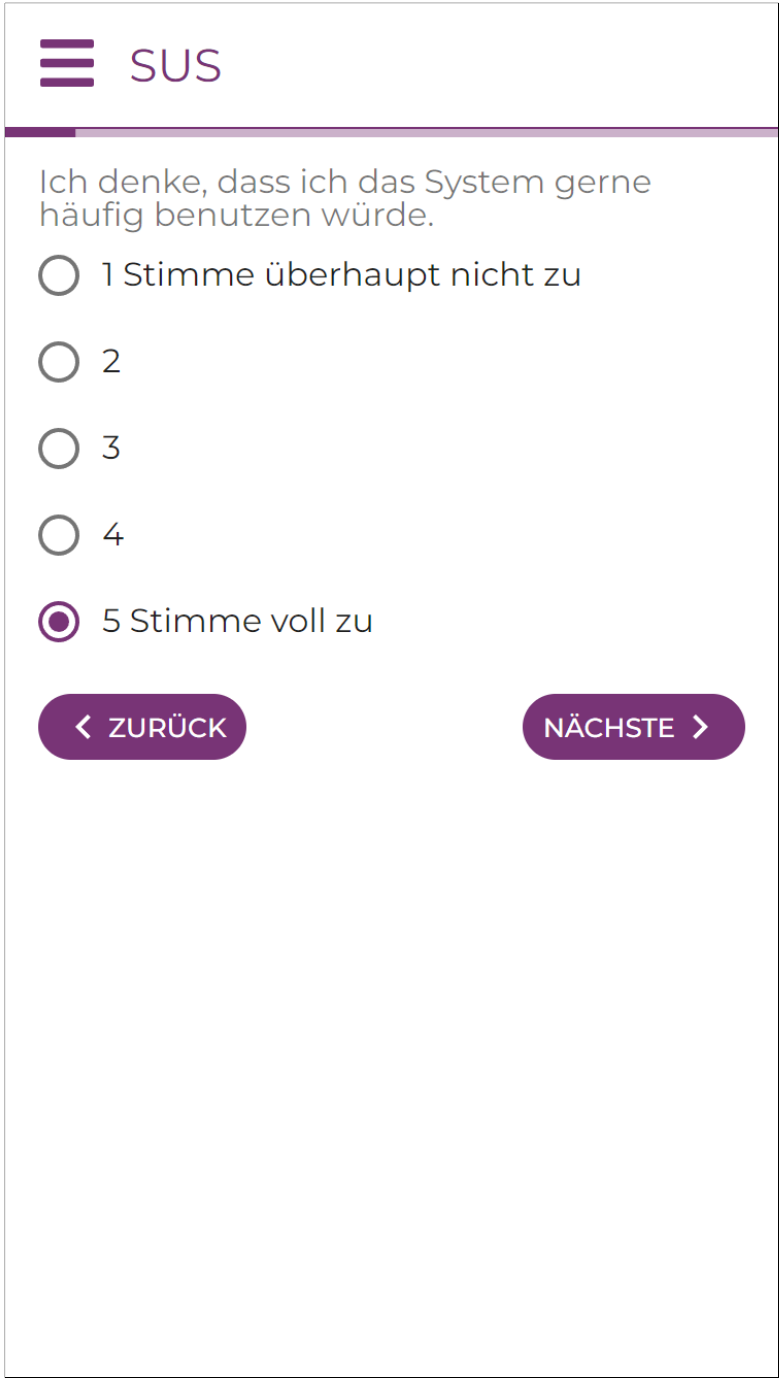

Questionnaires: UEQ

Introductory screen

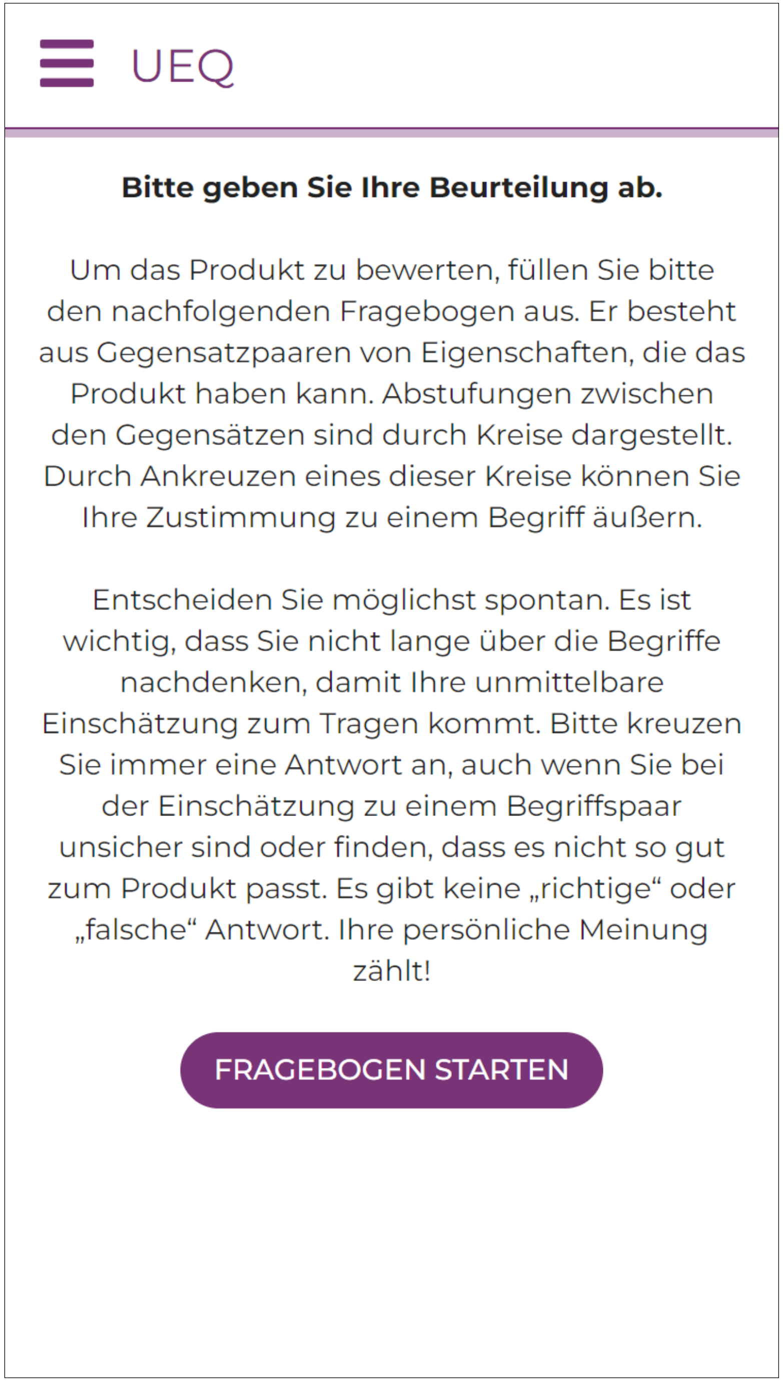

Questionnaires: UEQ

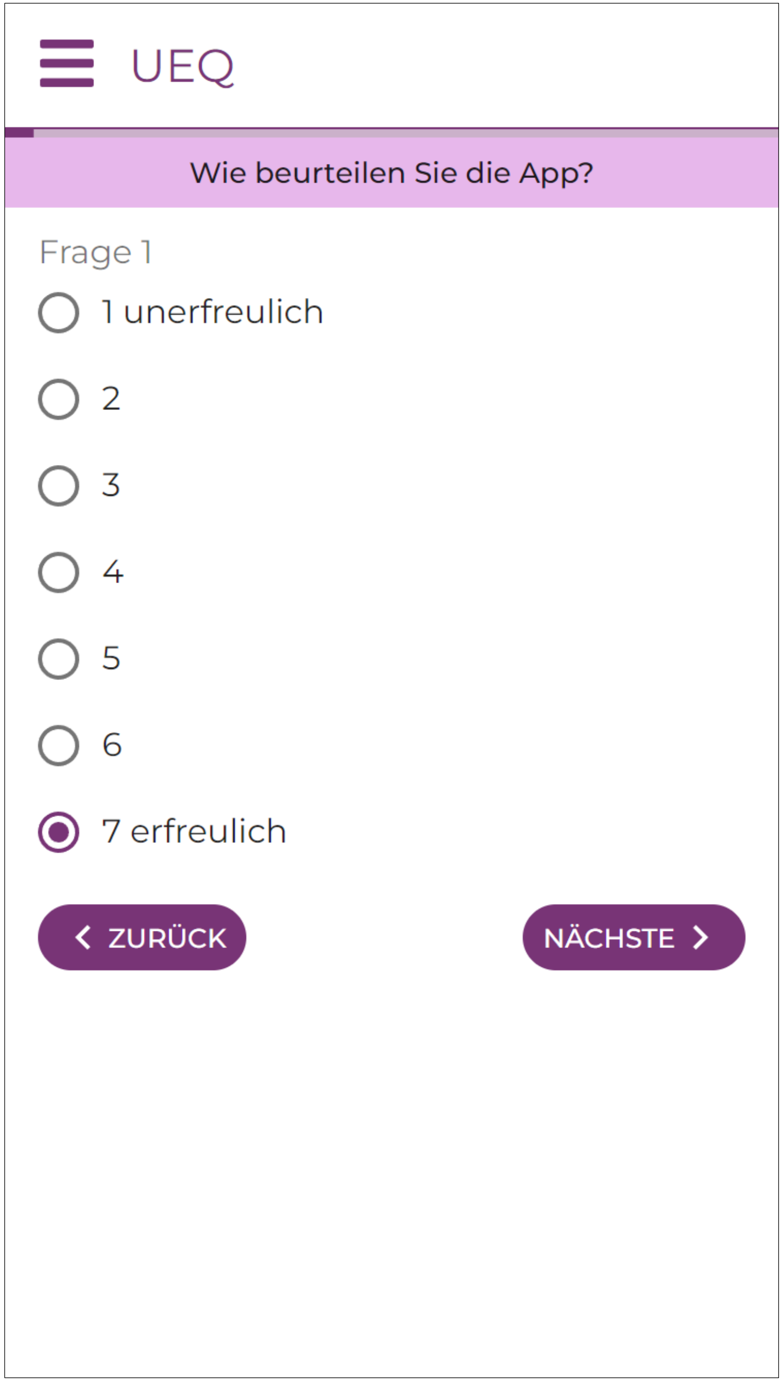

Exemplary question
