# Supplementary material for: Usability and Perception of a Wearable-Integrated Digital Maternity Record App in Germany: User Study
Source: JMIR Pediatr Parent. 2023 Dec 15;6:e50765. doi: 10.2196/50765 (PMC10750977; doi:10.2196/50765)
Supplement: Multimedia Appendix 3 [file pediatrics-v6-e50765-s003.pdf]

	Do you feel overwhelmed by the amount of information about your pregnancy?	*Have you actively searched for pregnancy information online? If so, how do you do it?*	*Are wearables something you would like to use? Would you like to have access to all your wearable data?*	*Would you prefer to hide all the wearable data and only receive a notification when there is an anomaly to act upon it?*	*Would you prefer to have fewer medical appointments and do more tests at home, recording the results by yourself?*	*How do you keep track of your pregnancy? Options: photo album, notebook with notes, folder with all the documentation*	*Would you share your data for research purposes? Under which conditions?*
P1	Yes. Prefers to trust the doctor.	Yes. Prefers to trust physician. Did online research for concrete question.	Yes, because of diabetes. All data is at one place and easily shared.	Be notified but be forced to perform a check-up with a physician.	Reduce the number of appointments but keep regular intervals. Depends on the individual pregnancy.	Does not keep a diary. It is good to have ultrasound images in the app.	Yes, with anonymity
P2	Yes. Short explanations are sufficient.	No. Only used books. Would search online if information is filtered and official.	No, unless required. Has privacy concerns.	Not convinced about either because unsure if app is trustworthy.	Is fine with the current number of appointments.	Not interested in saving other data like a journal or photos, unless she feels it is useful for pregnancy.	Would share the data anonymously, as she is aware of the difficulty of finding data for research.
P4	Yes. Still wants to see all the details.	Yes. Used Google and forums.	No, because of privacy issues and cyberchondria.	Would like to access to the values and have a recommendation to improve the situation.	Prefers going to the appointments. Feels safer to have a third person to check measurements.	Did not use any pregnancy app and does not think she would use it. Prefers photo albums in paper form.	Would not share it for a purpose.
P5	Yes, particularly if something unexpected happens.	Yes, but only for scientific papers, not blogs.	No, unless required, and would prefer the doctor to interpret the results.	Prefers to hide the data because she does not understand it. Prefers to only receive notifications if an anomaly occurs.	Likes the appointments, but sometimes did not even talk to anyone during these. These appointments could be omitted.	Not keen on the digitalization. Feels like the maternity record is a document for the doctors and not for her benefit.	Would share it for research under the condition that it cannot be linked to her.
P8	Yes. Prefers to trust the doctor.	Yes, but avoided Google and blogs, only relies on official sites.	No, only if there was a benefit.	Depends on the anomaly. Prefers not to have any notifications normally. This might be different if the notification/anomaly is important.	More convenient with less appointments if a long drive to the doctor's office is needed.	Does not use a pregnancy app. Thinks it is confusing and time consuming.	Already participated in such a program. Would share data if she knows about the study and approval from the ethics committee
P9	No, as no problems occurred in pregnancy.	Yes, but relied mainly on a mobile app to get information.	Yes, but only in emergency cases, such as a fall. Considers wearables to be "overcontrol" in general.	Prefers not to have a look at the data and leave reaction up to the physician.	Likes the appointments to have small questions. Would miss the appointments.	Used an app to track pregnancy.	Would participate in a study if it is anonymized.
P10	No, as no problems occurred in pregnancy.	Yes. Used Google because did not want to pay for other apps. Also read books.	Yes, if risk factors such as underweight or obesity exist.	Would not show the detailed data by default but start more general and then show more details.	Prefers going to the appointments and ask questions.	A journal with reminders might work. Social network is too much.	Has privacy concerns in her daily life but would not be worried about leaked information in her Mutterpass (maternity record).
P11	No. Likes to use apps to get information.	Yes, searched out of curiosity.	No, it is a lot to think about.	Would like notifications when there is very evident something is abnormal, but not in general.	The number of appointments is fine.	Used an app to track pregnancy.	Would share information unless it is very invasive.
P12	No. Feels safer with more information.	Yes, prefers official sites and avoids blogs.	No, maybe if the information is only visible the physician.	An excess of data could cause more anxiety, so none is better.	Is ok with the current number of appointments.	Used an app to track pregnancy.	Would share the data anonymously. Would not like that any doctor has access to her data.
P13	No. Feels safer with more information.	Yes, prefers official sites.	No, only if required.	Wearable data is important for her, she had high blood pressure and needed it. Useful to keep track of exercise during the day.	*(Question not asked)*	Used pregnancy apps for a diary and weight tracking.	Would do it with a clear data protection policy.
P14	No. Relies on physician.	Yes, mainly at the beginning of pregnancy.	No, only if required.	Would not like to see the detailed data in the default screen.	*(Question not asked)*	Thinks it is too much information and it is not keen to track all. Would only do it when there is a clinical reason.	Would only do if it is completely anonymized and if it follows regulatory recommendations.
